# Using a Co-Designed Digital Self-Management Program to Prepare Patients for Hip or Knee Replacement Surgery: Pragmatic Pilot Study

**DOI:** 10.2196/68286

**Published:** 2026-01-07

**Authors:** Elizabeth Horton, Hayley Wright, Andy Turner, Louise Moody, Lucy Aphramor, Anna Carlson, Hesam Ghiasvand, Shea Palmer

**Affiliations:** 1 Centre for Intelligent Healthcare, Coventry University, Priory Street, Coventry, CV1 5FB, United Kingdom; 2 Centre for Arts, Memory and Communities, Coventry University, Coventry, United Kingdom; 3 Centre for Agroecology, Water and Resilience, Coventry University, Coventry, United Kingdom; 4 Hope For The Community Community Interest Company, Enterprise Hub, Coventry, United Kingdom; 5 Centre for Healthcare and Communities, Coventry University, Coventry, United Kingdom; 6 School of Healthcare Sciences, Cardiff University, Cardiff, United Kingdom

**Keywords:** prehabilitation, self-management, peer support, digital platform, hip and knee arthroplasty, musculoskeletal

## Abstract

**Background:**

The aging population has resulted in more people living longer with musculoskeletal conditions who require hip and knee replacement surgery. Lengthening waiting lists continue to be a challenge for patients and health care services.

**Objective:**

This pragmatic study aimed to develop and test a digital self-management intervention (the HOPE [Help Overcome Problems Effectively] program) to better prepare patients waiting for hip and knee replacement surgery.

**Methods:**

The study used a pragmatic, pre-post with follow-up, single-arm design. All intervention and data collection components were delivered online. Patients were recruited from those on the waiting list for hip or knee surgery. Following iterative co-development of the intervention, the content was refined and optimized into a final version for testing. The resulting program was an 8-week intervention delivered via the HOPE 4 The Community (H4C) digital platform (powered by H4C). Data were collected at baseline (pre-HOPE program), 8 weeks (post-HOPE program), and 6-month follow-up. Patient-reported outcome measures related to preparation for surgery, quality of life, physical function, pain, mental well-being, self-efficacy, and physical activity. Resource usage data were collected to calculate health and social care costs. System Usability Scale data were collected post-HOPE program.

**Results:**

One hundred participants enrolled in the HOPE program. Of these, 57 (57%) consented to take part in the evaluation and returned the baseline questionnaire. Thirty-nine participants completed ≥5 of the 8 sessions and all surveys. Among the 25 participants who had surgery at 6 months, 23 (92%) felt better prepared due to the HOPE program. Median improvements in most outcomes were observed at 8 weeks, with several continuing to improve at 6 months. The Friedman test showed significant improvements over 6 months in self-efficacy (pain: *P*=.002; other symptoms: *P*<.01), pain (*P*=.04), health status (*P*=.02), and mental well-being (*P*=.01). No significant changes were noted in physical activity. While the early cost analysis did not reach statistical significance, it indicated potential cost savings from reduced patient interactions with health care professionals. Sixty-four percent (25/39) of participants had surgery, and this likely contributed in part to improvements in outcomes. System usability was rated above average (mean score 70.1, SD 15.9).

**Conclusions:**

The results are promising in relation to participants attending the HOPE program feeling better prepared for surgery. A fully powered efficacy and cost-effectiveness trial is needed to determine the contribution of the HOPE program to outcomes, over and above the contribution of surgery.

## Introduction

In the United Kingdom, an estimated 20.3 million people are affected by musculoskeletal conditions. These conditions account for 21% of the years lived with illness and disability [1]. The global prevalence of osteoarthritis is increasing, and if the trend continues, osteoarthritis will become one of the most prevalent diseases in populations from high-income countries in the coming decades [2]. The aging UK population is living longer with complex musculoskeletal conditions and comorbidities, causing increased demand on National Health Service (NHS) health and social care services [1], accounting for up to 30% of general practice consultations in England [3].

Lengthening waiting lists are particularly problematic in musculoskeletal medicine. A 2019 report found that in England alone, 726,000 people had severe hip osteoarthritis and 1.4 million people had severe knee osteoarthritis [4]. For those whose condition is severe, joint replacement surgery is the only option to alleviate pain and improve mobility and the ability to self-manage. Under the NHS constitution, 92% of patients should be treated within 18 weeks as part of the referral-to-treatment scheme. However, in 2019, nearly 4000 patients had been waiting for over 2 years for surgery [4], and more than 690,000 were on waiting lists in 2021 [5]. The COVID-19 pandemic had an unprecedented impact on secondary care orthopedic services, with a significant increase in waiting times for the majority of patients [5]. While on the waiting list, patients are likely to experience worsening pain, reduced mobility, increased anxiety, and deteriorating health, leading to greater demand for health and care services. In recognition of wait times, Versus Arthritis [5] and Arthritis Action [6] offer resources for self-management on their websites. By 2060, it is projected that the demand for hip and knee joint replacements in the United Kingdom will rise by nearly 40% from current levels, which will have significant implications for the health care system [7].

New ways of working are needed to optimize support for patients, maximize capacity, and mitigate risk. It is also important to address inequities: the COVID-19 pandemic foregrounded deep-rooted equality, diversity, and inclusion issues in relation to morbidity and mortality that are entangled with access to health care services. Inequities in treatment waiting time [8] for musculoskeletal services and in treatment outcomes [9] reflect this general picture and highlight the need for action. There is a need for holistic support among those waiting for hip and knee surgery in England. The NHS personalized care team recommends that patients on the waiting list should receive self-management support to “wait well” by undertaking prehabilitation. This support should empower patients through information, health coaching, and digital resources [10].

Prehabilitation is an effective way of improving perioperative outcomes through support to increase physical and mental resilience for surgery. Systematic reviews and meta-analyses have found some, generally low-quality evidence that prehabilitation improves a range of postoperative outcomes for patients undergoing hip and knee surgery, including function, pain, strength, and quality of life [11-13]. A more recent systematic review and meta-analysis specifically focused on the effects of digital prehabilitation in a range of musculoskeletal conditions awaiting surgery, including knee and hip replacements [14]. They found evidence that advanced technologies supported greater improvements in function pre- and post-operatively than standard care for knee and hip replacements. Greater improvements were also seen in preoperative pain, preoperative risk of falling, and postoperative stiffness. There was no evidence for spinal surgery or other conditions. However, few orthopedic prehabilitation interventions are digitally delivered, nor do they provide peer or emotional support, which is highly valued by many patients living with long-term conditions [11-13]. Indeed, a recent survey conducted in the United Kingdom [15] found that, although the vast majority of hospitals (97%) offered preoperative education, only 59% and 48% offered prehabilitation for knee and hip arthroplasty, respectively. Education was mainly delivered as a single talk supported by a booklet, and prehabilitation mainly as strengthening exercise, advice, and written information. Reported barriers included lack of facilities, funding, and staff. There was also a reported lack of robust evidence to support practice [15]. Across various surgical specialties, multimodal prehabilitation includes nutrition and psychological support alongside exercise training. There is some evidence of psychological factors improving postsurgical outcomes [13]. A systematic review and meta-analysis found low-quality evidence that psychological interventions have a positive effect on postsurgical anxiety and on mental components of quality of life [16].

In a review [17] of over 30 prehabilitation surgery schools in the United Kingdom and Ireland (these schools inform patients about what to expect and guide them on how to prepare physically and mentally to reduce postoperative risks of surgery), only 40% contained content to manage emotional well-being, and only 13% used digital apps. Further, many interventions were not underpinned by behavior change theory and techniques.

In 2022, Coventry University and its university spin-out social enterprise, H4C (HOPE [Help Overcome Problems Effectively] for The Community) interest company, developed a proof-of-concept digital intervention, called the Help Overcome Problems Effectively (HOPE) program, to help patients prepare for hip and knee surgery. The HOPE program for hip and knee patients shares the same underlying theoretical framework as other HOPE programs for long-term conditions offered by H4C, which have been taxonomized using the taxonomy of self-management support [18] and are described in detail in published papers [19-21]. All 14 digital versions of the HOPE program have been approved by the Quality Institute for Self-Management Education and Training for the provision of self-management structured education (QIS2020 and QIS2023 [22]) and certified by the Organisation for the Review of Care and Health Apps (ORCHA [23]), scoring 88% for Android and iOS (Apple Inc), and 86% for WebApp, indicating compliance with best practice in data security, professional assurance, usability, and accessibility.

The HOPE program for hip and knee patients combines evidence-based self-management content with a validated exercise program, incorporating a home exercise component tailored to individual needs and abilities, drawing from the work of Ageberg et al [24].

In 2023 H4C was awarded funding through the UK Research and Innovation Healthy Ageing Challenge Scaling Social Ventures competition to co-design and evaluate the HOPE program for hip and knee surgery patients. The funding competition was to support social enterprises in scaling products and services to support healthy aging and deliver social value.

The pragmatic, multimethod study aimed to optimize and evaluate the HOPE program to determine whether patients were better prepared for surgery. The study objectives included optimizing the HOPE program through co-design with stakeholders, implementing and testing the program with patients waiting for a joint replacement, and assessing their preparedness for surgery.

## Methods

### Study Design

This study used a co-design phase followed by a pragmatic, pre-post, with follow-up, single-arm intervention study. All intervention components and data collection were delivered online. This study is reported according to the CONSORT (Consolidated Standards of Reporting Trials) 2016 statement: extension for nonrandomized pilot trials [25]. CHERRIES (Checklist for Reporting Results of Internet E-Surveys) was used to guide the survey report [26]. All intervention and data collection activities took place online. All study data were collected online via questionnaires administered through Qualtrics Survey Software (Qualtrics).

### Co-Design Phase to Optimize the HOPE Program

Ten participants took part in the development activities, which included 3 online workshops. One workshop was undertaken with 6 patient participants waiting for a hip (n=3) or knee replacement surgery (n=3), who had completed an earlier proof-of-concept HOPE program (5 female participants, aged 60‐80 years). The purpose of the workshop was to explore their experiences and generate feedback on the HOPE program.

Two health professional workshops involving 4 NHS staff from our partner organizations were held to discuss referral pathways and useful resources for patients awaiting surgery. The roles of the professionals were Elective Recovery Lead, Team Lead Physiotherapist in Elective Orthopedics, Project Manager of a Musculoskeletal Clinical Program, and Senior Primary and Community Care Lead. Workshops and interviews were conducted online via Zoom (Zoom Video Communications, Inc) and MS Teams (Microsoft Corp) to allow for geographically dispersed participation.

### Development of the Exercise Program

The exercise program central to the intervention was based on the The Neuromuscular Exercise training program for patients with knee or hip osteoarthritis assigned for total joint replacement the neuromuscular exercise training program for patients with knee or hip osteoarthritis assigned for total joint replacement program [24,27], which was specifically developed for older patients with severe knee and hip osteoarthritis before having total joint replacement surgery. Only the exercises from the neuromuscular exercise training program for patients with knee or hip osteoarthritis assigned for total joint replacement program were adopted within the HOPE program. Those exercises have also been incorporated into the Good Life with osteoArthritis: Denmark program [28-30]. The exercises have been demonstrated to be safe, patients can successfully progress them, and they contribute to improvements in a range of outcomes, including symptoms, function, medication use, and sick leave. A range of video and visual resources had previously been developed to support the exercise components [31]. Following feedback from the co-design phase, new video resources were developed to illustrate how the exercises could be adapted within the home environment. Forty-three videos were filmed in a home setting (living room, bedroom, and kitchen), using home furniture (sofa, chair, and bed) and both exercise equipment and everyday household items as exercise props, with volunteers representing different ages and genders, and incorporating visual prompts and voiceover instructions. The exercises target major lower limb muscle groups and can be adapted to individual capabilities, with 3 difficulty levels and encouragement to alter repetitions and sets. Participants could build their own home-based exercise program by answering 6 questions about their ability (eg, if they can easily get on and off the floor) and equipment (eg, if they have a step they can use at home). An algorithm was then built to create their personalized exercise program from the 43 videos. Participants progressed up and down levels of difficulty at their own pace, monitored progress, and set exercise reminders. Participants could download their exercise record in PDF format to keep or share with a health care professional. Tips on creating a safe exercise space, as well as important information to mitigate any worries or injuries, were part of the program.

### The HOPE program: Intervention Content and Structure

The resulting program comprised 8 modules and was hosted on H4C’s digital platform, powered by H4C. The content comprised text, images, videos, downloadable documents, interactive activities (eg, quizzes, self-monitoring tools, and diaries), and discussion forums and messaging facilities. The digital content was released at set times over the 8 weeks but could be accessed at any time (asynchronous). Participants had the option to “fast-track” the content if they were due to have surgery during the 8 weeks.

Once accessed and viewed, the app content could be viewed offline, reducing the need for a data plan or high-quality internet connection. An analog print booklet was produced, containing the same content as the digital version of the HOPE program, for those who were digitally excluded and/or experienced low digital literacy.

### Pre-Post With Follow-Up Study

#### Participants

Broad eligibility criteria were used to ensure the study was as inclusive as possible and to provide ample opportunity for participation. Individuals were eligible if they were adults aged 18 years or older, lived in the South West of England in the United Kingdom, were currently on a waiting list for hip or knee replacement surgery, had access to the internet and a suitable device to engage with the intervention, and were able to interact with all materials provided as part of the intervention.

Patients interested in attending the HOPE program were referred to the study sign-up webpage through several routes. NHS South West referral sources included secondary care, primary care, and musculoskeletal clinics. Eligible participants were referred directly to H4C to enroll in the HOPE program and given the option to take part in the research study. Patients who chose to take part in the study were directed to the participant information sheet and consent form in Qualtrics Survey Software. Patients were informed that participation was voluntary and that their decision would not affect their quality of care.

We collected the following sociodemographic information: name, email address, gender, age, postcode, occupation and employment, and some details about their emotional health and their illness diagnosis, level of physical activity, health care visits, time on the waiting list, and date of surgery. Postcode data were used to calculate the English index of multiple deprivation (IMD [32]). IMD is an official measure of deprivation ranked from 1 (most deprived) to 10 (least deprived).

The questionnaire was administered through Qualtrics, using responsive and mobile-ready question formats. Adaptive questioning was used to conditionally display questions based on previous responses to reduce the number and complexity of the questions. Most pages contained between 1 and 6 items. Excluding the introduction, participant information sheet, and consent form, the survey was distributed over 14 pages. The responses were made mandatory to avoid missing data. The survey was not set up to allow participants to change their responses. The procedure, as outlined in the participant information sheet and survey structure, involved collecting identifiable information at registration—specifically, name and email address (rather than via technical means such as cookies or IP addresses)—which was then used by the research team to ensure each individual only completed the survey once per time point. Pre-HOPE program (baseline) questionnaires were completed during the period of July 6-13, 2023, for the first HOPE program and July 20-31, 2023, for the second HOPE program. Participants received a £60 (approximately US $80) electronic gift voucher for completion of all pre- and postprogram questionnaires. Participants were informed in the Patient Information Sheet how their data would be processed in accordance with the Data Protection Act 2018. Participation in the study was optional for patients who accessed the HOPE program.

#### The HOPE Program: Accessing and Completing the Program

Following completion of the pre-HOPE program survey, participants were given access to the HOPE program (start dates: July 13 or 27, 2023) through a personalized log-in link.

Throughout the program, participants were supported by 2 facilitators who were trained in line with Quality Institute for Self-Management Education and Training standards. The program content was organized into themed sessions across the 8 weeks, with an integrated tailored exercise program (described in the “Development of the Exercise Program” section above; Table 1 lists session content; refer to Multimedia Appendix 1 for a brief description of each session and screenshots of the intervention).

**Table 1.** Session content of the HOPE^a^ program.

Session	Session content
1	Instilling HOPE
2	Managing pain and fatigue
3	Stress and shifting your thinking
4	Communication
5	Sleep and mindfulness
6	Setbacks and hospital stay
7	Happiness and strengths
8	Moving on with HOPE

a HOPE: Help Overcome Problems Effectively.

#### Patient-Reported Outcome Measures

##### Surgery Preparation

At 6-month follow-up, participants were asked if they felt better prepared for surgery using the following question from the Patient Preparedness for Surgery questionnaire [33]: “Overall, I feel or felt (if I had surgery) prepared for my upcoming surgery*.*” There were 6 response options: strongly agree, agree, somewhat agree, somewhat disagree, disagree, and strongly disagree. Participants were also asked to provide reasons for their answers. Those who had surgery indicated whether they felt the HOPE program helped them prepare before surgery, after surgery, or both. Participants provided textual responses to explain why they agreed or disagreed that the HOPE program helped them prepare for surgery.

The following validated patient-reported outcome measures (PROMs) were collected at baseline, post-HOPE program (8 weeks), and 6-month follow-up via Qualtrics.

##### Short Warwick-Edinburgh Mental Well-Being Scale

The Short Warwick-Edinburgh Mental Well-Being Scale (SWEMWBS [34]) is a short version that assesses mental well-being within the adult population. The SWEMWBS uses 7 items from the full WEMWBS [35], which relate more to mental functioning than feelings. The 7 statements are positively worded, with 5 response categories ranging from “none of the time” to “all of the time.” Total scores range from 7 to 35, with higher scores indicating higher mental well-being. A change of one point or more on the SWEMWBS total score represents a minimally important level of change.

##### The EQ-5D Index and EQ-Visual Analogue Scale

The EQ-5D index [36] and the EQ-Visual Analogue Scale (EQ-VAS) are widely used measures of health status and health-related quality of life, respectively. The EQ-5D index assesses patients’ health state across 5 dimensions (self-care, mobility, anxiety and depression, usual activities, and pain and discomfort) that are weighted to provide a utility value based on a population tariff. Scores range from 0 (death) to 100 (perfect health). The EQ-VAS is a vertical rating scale for health, scored between 0 (worst imaginable health) and 100 (best imaginable health).

##### Western Ontario and McMaster Universities Arthritis Index

The Western Ontario and McMaster Universities Arthritis Index (WOMAC [37]) consists of 24 items divided into 3 subscales: Pain (5 items), Stiffness (2 items), and Physical Function (17 items). Items are scored on a scale of 0‐4, which corresponds to: None (0), Mild (1), Moderate (2), Severe (3), and Extreme (4). The scores for each subscale are summed, with possible score ranges of 0‐20 for Pain, 0‐8 for Stiffness, and 0‐68 for Physical Function. A sum of the scores for all 3 subscales gives a total WOMAC score (maximum 96). Higher scores indicate worse pain, stiffness, and functional limitations.

##### Arthritis Self-Efficacy Scale

The Arthritis Self-Efficacy Scale (ASES [38]) measures a person’s confidence to self-manage their arthritis symptoms and consists of 2 subscales: Pain (5 items) and Other Symptoms (6 items). Items are scored from 1 (very uncertain) to 10 (very certain). The scores for each subscale are summed, with a possible score range of 10‐50 for Pain and 10‐60 for Other Symptoms. Higher scores indicate higher self-efficacy.

##### International Physical Activity Questionnaire–Short Form

The International Physical Activity Questionnaire–Short Form (IPAQ-SF [39]) assesses physical activity undertaken across a comprehensive set of domains including: (1) leisure-time physical activity, (2) domestic and gardening (yard) activities, (3) work-related physical activity, and (4) transport-related physical activity. The items are structured to provide separate scores on walking, moderate-intensity, and vigorous-intensity activity, as well as a combined total score to describe the overall level of activity. Computation of the total score requires summation of the duration (in minutes) and frequency (days) of walking, moderate-intensity, and vigorous-intensity activity. The IPAQ-SF scoring protocol assigns the following metabolic equivalent of task (MET) values to walking, moderate, and vigorous-intensity activity: 3.3 METs, 4.0 METs, and 8.0 METs, respectively. Participants are considered to have met Centers for Disease Control and Prevention and American College of Sports Medicine physical activity recommendations if they reported at least 150 minutes per week of walking, moderate, or vigorous intensity physical activity.

##### Numerical Pain Rating Scale

The Numerical Rating Scale (NPRS)-11 [40] is an 11-point scale for self-report of pain. It is the most commonly used unidimensional pain scale. The respondent selects a whole number (integers 0‐10) that best reflects the intensity (or other quality, if requested) of their pain. The anchors are 0=no pain and 10=worst possible pain (there are various wordings of the upper anchor).

### HOPE Program Usability and User Engagement

#### Usability

The usability of the system was assessed by the System Usability Scale (SUS [41]), which was embedded in the last session of the HOPE program. It was optional for participants to complete. The SUS uses a 5-point Likert scale ranging from 1=strongly disagree to 5=strongly agree across 10 items. Odd-numbered questions (1, 3, 5, 7, and 9) generate a positive response. Even-numbered questions (2, 4, 6, 8, and 10) generate a negative response, which must be inverted. All the points added up together could gain a maximum of 40, thus the multiplication by 2.5 to make the scale out of 100. A total score of ≥68 is considered above-average usability.

#### User Engagement

The intervention platform collected user engagement data. For this study, we report the number of sessions completed, the number of participants who used the personalized exercise program, and the most commonly bookmarked content or activities.

### Sample Size

This pragmatic study enrolled participants from an opportunity sample (n=100) comprising eligible candidates. Potential participants received an email containing a link to the study website hosted by Qualtrics. Here, participants were required to review the digital Participant Information Sheet, provide digital consent, and complete the digital questionnaires.

### Analytical Methods

Data relating to sociodemographic characteristics and outcome measures were collated and presented descriptively at the group level. Outcome data were mostly ordinal and nonnormally distributed, so descriptive data were limited to frequencies (and proportions) and medians (and IQRs). While the study was not powered to detect statistically significant changes in outcomes between time points, nonparametric Friedman and post hoc Wilcoxon signed-rank tests were used to explore changes over time between baseline, post-HOPE program (8 weeks), and 6-month follow-up. All analyses were performed using IBM SPSS (version 28). The level of statistical significance was set at *P*<.05. Textual responses to the question about surgery preparedness at the 6-month follow-up survey were summarized to illustrate the quantitative findings.

Given this was a feasibility study with complete-case analysis as the prespecified approach, we focused on participants who engaged with ≥5 sessions and completed follow-ups. This decision was made because (1) the primary aim was assessing intervention feasibility and acceptability under optimal conditions, (2) minimal data were available from noncompleters (only 4/15 provided follow-ups), and (3) high follow-up rates among completers (98% at 8 weeks and 93% at 6 months) reduced concerns about attrition bias. Future efficacy trials will use intention-to-treat (ITT) analysis.

### Resource Usage

An early cost-impact analysis evaluated the change in costs associated with patients’ appointments and visits with NHS England to understand the potential cost impact of the program and assess whether it could be expanded into a broader study. These data were captured via the Qualtrics survey at baseline, post-HOPE program (8 weeks), and 6-month follow-up.

The economic analysis focused on changes in the number of interactions patients had with NHS health and social care staff, measured by appointments and visits. A decision model was developed using parameters from a before-and-after analysis, literature review, and incorporating assumptions. The mean values, associated SEs, and assumptions populated the model, detailed in Multimedia Appendix 2. The total cost impact was calculated from the NHS personal and social care perspective, both per patient and per patient per week.

Costs associated with interaction changes were evaluated at 8 weeks and 6 months compared to baseline using unit costs from the Unit Costs of Health and Social Care report by the Personal Social Services Research Unit at the University of Kent [42] and the NHS National Tariff [43]. A probabilistic sensitivity analysis explored uncertainty around the results.

### Ethical Considerations

The user requirements research undertaken by Coventry University received ethical approval from the Coventry University Research Ethics Committee (P151751). The research and evaluation activity has also received approval from Coventry University (P106036) and, as an amendment to a preexisting HOPE evaluation, from the Health Research Authority and Health and Care Research Wales (Integrated Research Applications System, project ID 283172).

## Results

### Co-Design Phase Adaptations

Adaptations to the intervention, as an outcome of patient and health professionals' feedback, were as follows: Adaptations suggested by patients were (1) guidance on how to adjust the exercises to meet individual needs and capabilities; (2) a broader range of additional activities to try, for example, pool-based exercises; (3) reassurance for people who may struggle to keep up with the program; (4) information to challenge misinformation, controversies, and conflicting advice; and (5) clearer guidance on how to access some features, for example, messaging functions.

Adaptations suggested by health professionals were (1) reminders and nudges to make healthy changes and prevent deconditioning, (2) long-term access to information for use postoperatively, (3) adjustment of exercises to cater to different abilities and comorbidities, (4) program certification to demonstrate credibility, and (5) reference to the expert input that informed the program content.

### Participants

One hundred participants enrolled in the 2 HOPE programs (HOPE 1: n=59 and HOPE 2: n=41). Of these, 57 (57%) consented to take part in the evaluation and returned the baseline questionnaire (n=39, HOPE program 1 and n=18, HOPE program 2). Forty-one participants returned follow-up questionnaires at 8 weeks (41/57, 71.9%), and 39 participants returned questionnaires at 6-month follow-up (39/57, 68.4%). Forty-two participants (42/57, 73.7%) accessed ≥5 of the 8 sessions and were considered program completers.

**Figure 1.**
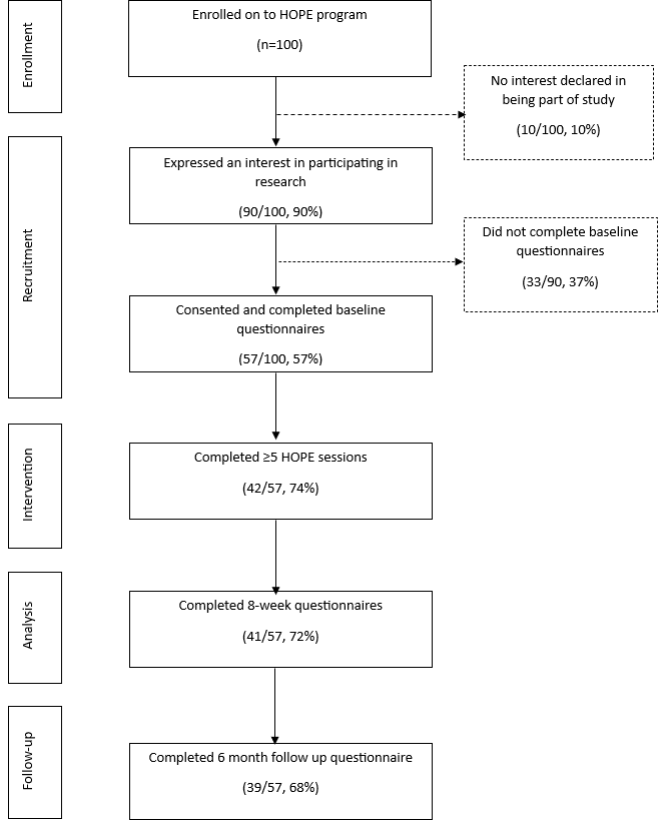
Flow of participants through the study. HOPE: Help Overcome Problems Effectively.

Almost all of the HOPE program completers (41/42, 98%) returned follow-up questionnaires at 8 weeks, and 39 (93%) returned questionnaires at 6-month follow-up (Figure 1). HOPE program completers who returned both questionnaires (39/42, 93%) were included in the primary analysis. There was no missing outcome data, as these fields were required during questionnaire completion.

Participant characteristics are presented in Table 2 and are similar in the total sample (n=57) and completers (n=39). All completer participants (n=39) identified as White-English, Welsh, Scottish, Northern Irish, or British ethnicity and described English as their first language (all 39/39, 100%). One-third of participants were male (13/39, 33%) and two-thirds were female (26/39, 67%). Age was only reported by 21 (54%) participants, with a median age of 66.0 (IQR 63.0-69.5) years. The majority of participants were retired (23/39, 59%). A third of participants (13/39, 33%) were listed for hip replacement surgery, and two-thirds (26/39, 67%) for knee replacement surgery. The median IMD was 7.00 (IQR 2.5-13) and the median time on the waiting list for surgery was 6.00 (IQR 2-12) months.

**Table 2.** Participant baseline characteristics of completers (n=39) and total sample (N=57).

Characteristic	Completers (n=39)	Total sample (N=57)
Gender, n (%)		
Male	13 (33)	20 (35)
Female	26 (67)	36 (63)
Not specified	0 (0)	1 (2)
Age (years), median (IQR)	66 (63-69.5)	66 (63-69.5)
Ethnicity, n (%)		
White-English, Welsh, Scottish, Northern Irish, or British	39 (100)	56 (98)
Black, African, or Caribbean	0 (0)	1 (2)
Disability, n (%)		
Mental health condition (long-term)	5 (13)	7 (12)
Blind or partially sighted	1 (3)	1 (2)
Hard of hearing or deaf	0 (0)	1 (2)
Long-term illness or health condition (lasting more than 12 months or terminal)	4 (10)	7 (12)
Mobility impairment	24 (62)	32 (56)
Employment, n (%)		
In paid work: full-time	4 (10)	8 (14)
In paid work: part-time	4 (10)	8 (14)
Retired	23 (59)	31 (54)
Not in paid work	8 (21)	10 (18)
Not in paid work due to hip or knee condition?	8 (21)	10 (18)
Index of multiple deprivation, median (IQR)	7 (2.25)	7 (2)
Joint replacement, n (%)		
Hip	13 (33)	22 (39)
Knee	26 (67)	35 (61)
Waiting time (months), median (IQR)	6 (2-12)	7 (2.5-13)

Twenty-four out of 39 (62%) participants considered themselves to have a disability, with 9 (23%) participants reporting that day-to-day activities were “limited a little,” and 15 (39%) reporting that activities were “limited a lot.” Seven (18%) participants reported more than one specific type of disability (refer to Table 2).

### User Engagement

Just over half of all participants completed all 8 sessions (30/57, 53%), with 6 participants completing 7 sessions (6/57, 11%), 1 completing 6 sessions (1/57, 2%), and a further 5 participants completing 5 sessions (5/57, 9%). Forty-nine out of 57 (86%) participants used the personalized exercise program. The top 5 bookmarked content or activities were (1) exercise program, (2) relaxed breathing, (3) mindfulness meditation, (4) compassionate approach to pain, and (5) cognitive diffusion activity.

### Patient-Reported Outcomes and Estimations

By the time of the 6-month follow-up, 25 out of 39 (64%) participants had already received their surgery. Of those who had their surgery, the majority (23/25; 92%) agreed with the statement: *“*As a result of attending the HOPE program, overall, I felt better prepared for my surgery.*”* Eight out of 23 (35%) participants selected “strongly agree*,”* 10 (44%) selected “agree*,”* and 5 (22%) selected “somewhat agree*.”* Of the 23 participants who agreed that they were better prepared, 16 (70%) felt better prepared in the presurgery period, 3 (13%) felt better prepared postsurgery, and 5 (17%) felt better prepared pre- and postsurgery.

Of those who had not yet had surgery, the majority (13/14, 93%) agreed with the statement: *“*As a result of attending the HOPE program, overall, I feel better prepared for my surgery.” Of these, 1 participant (1/14, 7%) selected “strongly agree*,*” 7 (n=7/14, 50%) selected “agree*,*” and 5 (5/23, 30%) selected “somewhat agree*.*”

All 39 participants who completed the 6-month follow-up questionnaire provided reasons why they agreed or disagreed that the HOPE program helped them prepare for surgery. The findings are presented under 4 headings: personalized exercise, physical and mental preparation, peer support, and nothing new. Participant ID numbers 1‐14 are those that were still waiting for surgery at 6-month follow-up, and IDs 15-39 are participants who had undergone surgery. No harms or unintended consequences were reported during the study.

#### Personalized Exercise

The program offered exercises that helped patients improve their physical condition and overall preparedness for surgery.

*Better exercised and with better muscle definition.*

[ID19]

*It gave me some exercises to prepare for surgery.*

[ID29]

*It encouraged me to do the preparation exercises and helped lift my mood when needed.*

[ID20]

#### Physical and Mental Preparation

Patients found the program beneficial for preparing both physically and mentally for surgery. It provided information and insight about what to expect before and after surgery, helping to manage pain, reduce anxiety, increase hope, and plan for the future.

*The program gave me an insight into what to expect during and after the procedure.*

[ID18]

*I found the information useful and the relaxation techniques particularly helpful.*

[ID38]

*The information given was clear about the future after the operation.*

[ID7]

*I feel I manage pain better even if it becomes more painful.*

[ID8]

*I knew so much about what to expect, and I learned techniques to calm any anxiety.*

[ID33]

#### Peer Support

Connecting with others who have arthritis and are waiting for surgery made patients feel less isolated. The program offered a platform for discussing shared challenges, such as surgery delays and recovery expectations, fostering a sense of community among participants. Participants valued the emotional support they received through the program. Sharing experiences with others who were undergoing similar surgeries provided comfort, while insights into the surgical process helped ease fears.

*Hearing what other arthritis sufferers are going through made you feel that you are not alone in dealing with the pain.*

[ID1]

*The HOPE program gave me the opportunity to share my thoughts/fears with others who had either had their joint surgery or were waiting to undergo it.*

[ID17]

#### Nothing New

A few participants found that the program covered what they already knew or that they already had a positive mindset.

*I haven’t found out anything new.*

[ID9]

*I already had a very positive view of how to deal with the issues arising from my arthritis.*

[ID10]

### Usability

Only 16 participants completed the optional SUS. Participants reported a mean SUS score of 70.1 (SD 15.9; range 50‐95). The 10-item frequency response data are provided in Multimedia Appendix 3. Compared with the 23 participants who did not complete the SUS, the 16 SUS completers were younger (median age of 64, IQR 8 vs 67, IQR 6 years; sample size n=8 and n=13, respectively), included a higher proportion of males (44% vs 26%), and more knee surgery patients (75% vs 60%) with a mobility impairment (69% vs 50%). Other patient characteristics were broadly similar. On average, the 16 completers had slightly lower disease severity: total WOMAC median 49 (IQR 23) versus 53 (IQR 17).

Table 3 summarizes the patient-reported outcomes at baseline, 8 weeks (post-HOPE program), and 6-month follow-up. Median values suggested potential improvements in many outcome measures at the end of the HOPE program (8 weeks). There were sustained improvements in median values for several outcomes at 6 months.

**Table 3.** Summary of baseline, post–Help Overcome Problems Effectively (HOPE) program (8 weeks), and 6-month follow-up outcomes (n=39).

Outcome variable	Baseline, median (IQR)	8 weeks, median (IQR)	*P* value, Wilcoxon test (baseline to 8 week)	6 months, median (IQR)	*P* value, Friedman test
Arthritis Self-Efficacy Scale (ASES)					
Confidence to manage pain (1‐10, ↑=better)	3.8 (2.0-5.6)	4.6 (3.6-5.4)	.07	5.6 (3.8-8.2)	.002^a^
Confidence to manage other symptoms (1‐10, ↑=better)	4.5 (2.5-5.5)	5.0 (4.2-6.5)	.001^a^	6.5 (4.2-8.3)	<.001^a^
Western Ontario and McMaster Osteoarthritis Index (WOMAC)					
Pain (0‐20, ↑=worse)	10.0 (8.0-12.0)	10.0 (7.0-13.0)	.19	8.0 (5.0-12.0)	.0^a^4
Stiffness (0‐8, ↑=worse)	4.0 (4.0-5.0)	4.0 (4.0-5.0)	.92	4.0 (2.0-5.0)	.11
Physical functioning (0‐68, ↑=worse)	35.0 (26.0-41.0)	34.0 (24.0-42.0)	.44	29.0 (10.0-42.0)	.07
Total (0‐96, ↑=worse)	49.0 (40.0-58.0)	48.0 (33.0-59.0)	.38	39.0 (19.0-60.0)	.09
Numerical Pain Rating Scale (NPRS)					
Pain (0‐10, ↑=worse)	6.0 (5.0-7.0)	5.0 (4.0-7.0)	.19	5.0 (2.0-6.0)	.002^a^
EQ-5D					
Quality of Life (EQ-VAS^c^; 0‐100, ↑=better)	58.0 (35.0-80.0)	60.0 (35.0-80.0)	.37	70.0 (45.0-85.0)	.05
Health status (EQ-Index; 0‐1, ↑=better)	0.62 (0.30-0.74)	0.60 (0.23-0.78)	.54	0.75 (0.30-0.83)	.02^a^
Short Warwick-Edinburgh Mental Well-Being Scale (SWEMWBS)					
Mental well-being (SWEMWBS; 5‐35, ↑=better)	25.0 (21.0-28.0)	25.0 (22.0-28.0)	.86	27.0 (23.0-29.0)	.01^a^
International Physical Activity Questionnaire–Short Form (IPAQ-SF)					
Total (MET-min/wk, ↑=better)	2340 (393-7464)	2628 (480-6152)	.98	2079 (306-5988)	.55
Sitting time (min/d, ↑=worse)	360 (285-480)	360 (181-540)	.41	300 (240-480)	.15
Inactive (<600 MET^b^-min/wk), n (%)	12 (30.8)	10 (25.6)	—^d^	12 (30.8)	—^d^

a Statistically significant *P*<05. Data is for participants who participated in ≥5 sessions and completed both follow-up questionnaires (n=39).

b MET: metabolic equivalent.

c EQ-VAS: EQ-Visual Analogue Scale.

d Not applicable.

The Friedman test indicated several potential improvements across the 6 months. Median scores for ASES pain were 3.8 (IQR 2.0-5.6), 4.6 (IQR 3.6-5.4), and 5.6 (IQR 3.8-8.2) at baseline, 8 weeks, and 6-month follow-up, respectively (*P*=.002); ASES other symptoms: 4.5 (IQR 2.5-5.5), 5.0 (IQR 4.2-6.5), and 6.5 (IQR 4.2-8.3*; P*<.001); WOMAC pain: 10.0 (IQR 8.0-12.0), 10.0 (IQR 7.0-13.0), and 8.0 (IQR 5.0-12.0*; P*=.04); NRPS: 6.0 (IQR 5.0-7.0), 5.0 (IQR 4.0-7.0), and 5.0 (IQR 2.0-6.0*; P*=.002); EQ-index: 0.62 (IQR 0.3-0.74), 0.60 (IQR 0.23-0.78), and 0.75 (IQR 0.30-0.83*; P*=.02); and SWEMWBS: 25.0 (IQR 21.0-28.0), 25 (IQR 22.0-28.0), and 27.0 (IQR 23.0-29.0*; P*=.01). Separate Wilcoxon tests at 8 weeks (immediately following the end of the HOPE program) found that only ASES other symptoms was statistically significant (*P*=.001; refer to Table 3).

### Ancillary Analyses

#### Assessment of Bias: Program Completers Versus Program Noncompleters at Baseline

A total of 15 participants were categorized as noncompleters of the Hope program (ie, completing <5 of 8 sessions). Only 4 (27%) of these participants returned both follow-up questionnaires, which was insufficient for meaningful analysis. Therefore, bias assessment was conducted using baseline data only. Potential differences at baseline between noncompleters (<5 sessions; n=15) and completers (≥5 sessions; n=42) were explored using descriptive statistics. There were no obvious differences between noncompleters and completers in age (median 65.50, IQR 64-72.75 vs median 66.00, IQR 63.0-69.0 years, respectively) or IMD (median 7.00, IQR 5.0-8.0 vs median 7.00, IQR 6.0-8.25). There were slight differences between noncompleters and completers in gender (male: 50% vs 31%), ethnicity (White: 93% vs 100%), disability (yes: 53% vs 62%), employment (in paid work—full time and part time: 54% vs 20%), joint replacement (knee: 47% vs 67%), and waiting time (7.00, IQR 4.0-16.0 vs 6.00, IQR 2.0-13.0 months). On average, noncompleters had slightly greater disease severity. For example, noncompleters reported more pain (NPRS: median 7.00, IQR 6.0-8.0 vs median 6.00, IQR 5.0-7.0, respectively), had a higher total WOMAC score (median 60.00, IQR 40.0-71.0 vs median 49.00, IQR 38.75-58.25), and a lower EQ-5D index value (median 0.45, IQR 0.18-0.73 and median 0.636, IQR 0.29-0.74).

#### Impact of Surgery on Outcomes

To understand the potential impact of surgery on outcomes, an exploratory descriptive comparison between those who had and had not received surgery at 6 months was made (data presented in Multimedia Appendix 4). This comparison was only based on the ASES data, since improvements in this outcome were statistically significant at both 8 weeks and 6 months. The results show that those who had received surgery at 6 months had larger median improvements in self-efficacy (for both pain and other symptoms). Those who had not had surgery showed marginal improvements in self-efficacy for pain and for other symptoms at 8 weeks. These were maintained at 6 months for pain self-efficacy but not for other symptoms.

### Health Care Resource Usage

The component of the study that focused on resource use for this early cost-impact analysis had a sample size of 39 patients, who completed the web-based questionnaire at all 3 time points: baseline, after 8 weeks, and after 6 months. Of these, 25 patients had their surgical intervention within the period covered (ie, within 6 months) and were therefore excluded from the analysis, leaving a total sample size of 14 analyzed. Results are provided in Table 4 (total cost-impact per patient) and Table 5 (cost-impact per patient per week). Cost-impact per patient per week evaluation revealed overall cost savings over 8 weeks as well as over 6 months, but this failed to reach statistical significance. Face-to-face general practitioner interactions at the 6-month interval showed a statistically significant change. Further details of the economic analysis are provided in Multimedia Appendix 2.

**Table 4.** Total cost impact per patient (£per week).

Cost category^a^	Cost change from baseline to 8 weeks, mean (95% CI)	Cost change from baseline to 6 months, mean (95% CI)
Face-to-face visit with a physiotherapist	12.85 (–3.80 to 36.02)	–8.64 (–76.34 to 27.29)
Remote visit with GP^b^	–7.72 (–19.55 to 0.36)	0.22 (–3.38 to 4.07)
Face-to-face visit with GP	9.66 (–12.57 to 34.00)	14.09 (–5.39 to 36.29)
Face-to-face hospital visit	–7.46 (–29.65 to 10.52)	–8.91 (–38.35 to 10.84)
Total	7.34 (46.49 to –27.61)	–3.25 (46.10 to –87.02)

a Positive values correspond to cost savings.

b GP: general practitioner.

**Table 5.** Cost impact per patient (£ per week).

Cost category^a^	Cost change from baseline to 8 weeks, mean (95% CI)	Cost change from baseline to 6 months, mean (95% CI)
Face-to-face visit with physiotherapist	1.61 (–0.48 to 4.5)	0.69 (–3.34 to 3.94)
Remote visit with ^c^GP	–0.96 (–2.44 to 0.04)	0.20 (–0.1 to 0.63)
Face-to-face visit with GP	1.21 (–1.57 to 4.25)	2.45 (0.50 to 5.08)^b^
Face-to-face hospital visit	–0.93 (–3.71 to 1.31)	–0.03 (–1.85 to 1.82)
Total	0.92 (–3.45 to 5.81)	3.31 (–1.93 to 8.12)

a Positive values correspond to cost savings.

b Statistically significant (*P*<.05).

c GP: general pratitioner.

### Sample Size Calculation for Future Trial

Data collected as part of the current evaluation were used to inform likely sample sizes for future studies in this area. This sample size calculation was based on ASES-8 data. Unfortunately, the minimum clinically important difference of the ASES-8 is unknown [44]. However, it is sensitive to change, with an effect size of 0.31 previously reported for the ASES-8 following interdisciplinary group therapy for fibromyalgia [45]. Moderate effect sizes of this magnitude are common for conservative interventions in musculoskeletal conditions. In this pilot study, the mean and SD values for ASES pain and ASES other symptoms at baseline were 3.98 (SD 1.93) and 4.29 (SD 2.08), respectively. Assuming a 1-tailed hypothesis, an effect size of 0.3, α*=*.05, 90% power, and a 1:1 allocation ratio, 191 participants would be required in each group (N=382) to detect a ≥0.58-point difference in ASES pain and ≥0.62-point difference in ASES other symptoms.

## Discussion

### Principal Findings

This study evaluated the HOPE program, a digital self-management intervention designed to support patients awaiting hip and knee replacement surgery. Results from 39 completers suggested potential improvements in self-efficacy, pain, health status, and mental well-being over 6 months. Most participants felt better prepared for surgery, and the program was rated above average for usability (mean SUS score 70.1).

Participant feedback revealed some key areas that underscore the program’s potential usefulness. Some participants appreciated the targeted exercises that improved their physical and mental readiness for surgery. The program provided comprehensive information about the surgical process, helping patients manage pain, reduce anxiety, and plan for the future. Studies have shown that patients have difficulties remembering information immediately after deciding to undergo surgery [46]. Having access to digital information, which can be regularly and quickly updated with evidence-based information, is a useful resource for patients. By fostering a sense of community, the program helped some participants connect with others facing similar challenges. However, some participants noted that the program offered nothing new, as they already enjoyed a positive mindset or previous knowledge.

The demographic profile of completers (median age 66, IQR 63-69.5 years; 100% White; and 66.7% female) was almost identical to a recent UK study, which found that digital health coaching delivered to patients waiting for lower limb arthroplasty improved patient activation and reduced length of hospital stay [47]. It should be noted that noncompleters of the program were more likely to be male, in paid employment, and awaiting a hip replacement.

Engagement with the HOPE program was high, with 73.7% (42/57) of participants attending ≥5 of 8 sessions. Follow-up and engagement rates were lower when based on the 100 participants who enrolled: 39% (39/100) completed the 6-month follow-up questionnaires, and 42% (42/100) who completed ≥5 sessions. Among those who completed all study procedures, 93% (39/42) engaged with the program.

A recent national digital attitudes and behavior survey conducted in the United Kingdom by ORCHA in 2023 described the willingness of older respondents to use digital apps for self-monitoring, symptom tracking, and managing recovery [48].

At the 6-month follow-up, nearly two-thirds (25/39, 64%) of participants had undergone surgery. More than 90% (23/25) of these participants agreed that the program helped them prepare better for surgery. Statistically significant median improvements in most PROMs were evident at the end of the HOPE program, and several scores continued to improve at 6-month follow-up, including self-efficacy, pain, health status, and mental well-being. The exercise program was the most bookmarked page, and despite the majority of participants (49/57, 86%) starting the personalized exercise program, there were no improvements in time spent sitting or in the proportion of participants classified as inactive. The exercise program may require greater input from facilitators to encourage optimum engagement. Research shows that exercise supervision involving trained physical therapists improves compliance with exercises, especially in older adults [13,49]. Alternatively, it may be that the IPAQ-SF lacks sensitivity to adequately assess physical activity [39]. More objective measures of physical activity, such as accelerometry, could be considered in future research.

The high number of participants undergoing surgery makes it challenging to attribute potential improvements in PROMs to either intervention. In their systematic review and meta-analysis, Punnoose et al [13] showed that variability in surgical procedures can influence postoperative recovery; therefore, postsurgical improvements cannot be attributable solely to prehabilitation. Owing to the often degenerative nature of musculoskeletal conditions, potential improvements in PROMs in this study were not anticipated a priori. Rather, it was hypothesized that attending the HOPE program would slow the rate of decline through the acquisition of effective self-management and coping strategies. Thus, the observed trend for median improvements across the majority of PROMs is encouraging.

### Resource Usage

This early cost analysis suggests that the HOPE program may lead to a reduction in patient interactions with care professionals at both 8 weeks and 6 months. However, the small sample size results in wide CIs, which limits the reliability of these findings and affirms the need for further studies to assess the cost-effectiveness of the program. Despite this limitation, the initial results highlight the potential for the HOPE program to offer cost-saving benefits at a societal level.

### Strengths and Limitations

A strength of this real-world study was the inclusion of participants with lived experience at all stages of the project, providing input into the HOPE program intervention development process and follow-up feedback to optimize it for further studies. The majority of participants started the exercise program, which is a cornerstone of prehabilitation. Other strengths include the use of validated PROMs, high levels of engagement with the intervention, and good survey completion rates at 6 months. This version of the HOPE program was rapidly developed and deployed by adding new musculoskeletal content to an existing taxonomized evidence-based intervention. Some of the health professionals involved in the co-design workshops suggested that patients needing only conservative management and not requiring surgery would also benefit from the program. Our co-creation and intervention development process could develop and test a program for these patients and for other groups of nonorthopedic presurgery patients. The powered by H4C platform currently hosts more than 15 digital self-management and health interventions. Using a single platform to deliver multiple interventions and modules offers several advantages for funders, researchers, health care providers, and patients. Many patients live with comorbid conditions requiring diverse information and self-management techniques. Platform delivery can incorporate and streamline self-management support. Torous and Vaidyam [50] asserted that “instead of a plethora of apps, there is a need for a few that meet the needs of many.” Drawing on successful examples from the automobile, space, and clean energy sector, Ansar and Flyvbjerg [51] outline the benefits of platforms over one-off designs, such as repeatability, extendibility, absorptive and adaptive capacity, resulting in “faster, better, cheaper” services and products. They concluded that sectors such as health “are ripe for a platform rethink.” Another strength of this application is the partnership between a social enterprise company and an academic institution. A recent Wellcome report [52] recommended that companies, including nonprofits, can be better at developing and scaling digital health solutions than university research groups.

The limitations of the study include the small number (16/39) of participants who completed the SUS. It is possible that these participants had a more positive user experience compared to those who did not complete the scale. Among the 39 study completers, most participants (>90%) agreed that the program helped them prepare better for surgery, and the textual responses supporting this question provided limited feedback. A broader set of feedback questions and/or postprogram qualitative interviews or focus groups analyzed using rigorous and transparent methods—with participants who did not complete the program—could elicit more critical or negative experiences.

The self-selecting nature of recruitment may have resulted in participants who were inherently more inclined to seek assistance or engage in self-help efforts.

Without a control group comparator, it is not possible to directly attribute any change in the PROMs to the HOPE program. It is important to note that many improvements were not statistically significant, and the statistical analyses performed were likely to be underpowered. Furthermore, a recent systematic review of hip arthroplasty prehabilitation interventions suggested that measures such as the WOMAC may not be the most appropriate measure to detect differences and suggest alternative objective measures such as the chair rise test, gait speed, or stair climbing [53]. That review also found that more than 8 weeks of prehabilitation was associated with improved outcomes, suggesting that future trials of the HOPE program should consider extending the length of the intervention. While our completer analysis provides valuable proof-of-concept data, it limits generalizability to real-world implementation, where attrition is typically higher. The baseline differences between completers and noncompleters suggest our effect estimates may be optimistic. Future randomized controlled trials (RCTs) should combine ITT and per-protocol analyses to distinguish efficacy from effectiveness.

Separating the effects of the intervention from the effects of surgery is problematic. The ancillary analysis of the ASES data (refer to Table 4) suggests that surgery was probably a major contributor to improvements in self-efficacy at 6 months. This is not surprising, given that the excellent outcomes of hip replacement surgery have led to the procedure being described by The Lancet as the “operation of the century” [54]. Approximately 96.2% and 90.8% of patients have previously reported satisfaction with their hip and knee replacement surgery, respectively [55].

A future definitive RCT should be appropriately powered to directly compare an intervention group (ie, the HOPE program) against an appropriate control group (ie, treatment as usual). Subgroup analysis should compare PROMs in those who have had, or are still awaiting, surgery at 6-month follow-up. Such a design would help to distinguish the effects of the intervention from the effects of surgery.

The baseline data show that program noncompleters (ie, those who completed <5 sessions) had slightly greater disease severity at baseline than program completers. Owing to limited follow-up data, it is not known whether these participants could not complete the program due to factors relating to their musculoskeletal condition, their experience of the program, or random intervening factors. Nonresponders were also more likely to be male, in paid employment, and awaiting a hip replacement. Such findings raise questions about how to engage people with greater disease severity and these sociodemographic characteristics in future support programs. Further research is needed to understand individual needs and how they change as disease and pain progress, and to determine how best to support individuals through targeted interventions.

In line with the wealth of other UK health care research studies, the participant sample in this study lacked diversity in terms of ethnicity and socioeconomic characteristics. The study sample reflects the demographics of NHS waiting lists and can be understood as a manifestation of structural inequalities. People living in the most deprived areas of the United Kingdom are more likely to require replacement surgery but less likely to receive it [56] and less likely to have good outcomes [57,58] compared with those living in the least deprived areas. This recurring finding underscores the need for research into the impact of structural barriers to self-management, which may, in turn, suggest the need for more options or a new paradigm approach. Health care interventions that disproportionately meet the needs of nonmarginalized groups embed injustice by widening health inequity. The earlier statement that no harm was reported during the study holds when “harm” is understood within the parameters of evidence-based medicine and its associated framework of biomedical ethics. However, when a framing such as distributive justice is applied, the intervention may be associated with unintended adverse consequences that emerge from and perpetuate ideologies such as structural racism and classism. Lack of attention to unintended harm linked to the lack of diversity in self-management research highlights the need for an expanded ethical framework informed by disability justice scholarship [59]. Recommendations from a recent report into musculoskeletal health inequalities in the United Kingdom included prioritizing surgery and self-management support for patients living in the most deprived areas [60]. More effort is required to understand the needs of and actively recruit these groups of participants in future self-management trials. A national digital attitudes and behavior survey conducted in the United Kingdom by ORCHA in 2021 [61] found that advocacy for digital health apps was highest among people of Black African heritage (89%), followed by Asian (80%), and then White (64%) respondents. Studies from the United States highlight the importance of recruiting low-income and ethnic minority participants, showing that these groups are more willing to attend [62] and engage more [63] with health interventions compared with White participants in higher-income groups. However, data from this study show that deprivation levels were similar between HOPE program completers and noncompleters.

### Conclusion

The results are promising in relation to the acceptability of a peer-supported self-management program for people awaiting hip or knee surgery. Overall, participants felt better prepared for surgery. Textual feedback was generally positive, and participants attributed improvements in their mental and physical well-being to techniques they learned in the HOPE program. However, comparing self-efficacy in those who had and had not received surgery suggests that surgery might have been a more important agent of change than the HOPE program. Overall, the study has demonstrated potential benefit and no evidence of harm or unintended consequences. A randomized controlled efficacy and cost-effectiveness trial design, involving a socioeconomically and ethnically representative sample, is required to delineate the effects attributable to the HOPE program, as opposed to effects of having surgery or natural variation in PROMs. While these preliminary results are promising, they require confirmation in a fully powered RCT using ITT analysis to account for real-world attrition patterns.

## Supplementary material

10.2196/68286 Multimedia Appendix 1 Intervention screenshot and session content.

10.2196/68286 Multimedia Appendix 2 Health economic evaluation analysis.

10.2196/68286 Multimedia Appendix 3 System Usability Scale scores.

10.2196/68286 Multimedia Appendix 4 Changes in Arthritis Self-Efficacy Scale.

10.2196/68286 Checklist 1 CHERRIES (Checklist for Reporting Results of Internet E-Surveys) checklist.

10.2196/68286 Checklist 2 CONSORT-EHEALTH checklist.

## Abbreviations

ASES: Arthritis Self-Efficacy Scale
CHERRIES: Checklist for Reporting Results of Internet E-Surveys
CONSORT: Consolidated Standards of Reporting Trials
EQ-VAS: EQ-Visual Analogue Scale
H4C: HOPE 4 The Community Interest Company
HOPE: Help Overcome Problems Effectively
IMD: index of multiple deprivation
IPAQ-SF: International Physical Activity Questionnaire–Short Form
ITT: intention-to-treat
MET: metabolic equivalent of task
NHS: National Health Service
NPRS: Numerical Pain Rating Scale
ORCHA: Organisation for the Review of Care and Health Apps
PROM: patient-reported outcome measure
RCT: randomized controlled trial
SUS: System Usability Scale
SWEMWBS: Short Warwick-Edinburgh Mental Well-Being Scale
WOMAC: Western Ontario and McMaster Universities Arthritis Index
