# Supplementary material for: Using a Co-Designed Digital Self-Management Program to Prepare Patients for Hip or Knee Replacement Surgery: Pragmatic Pilot Study
Source: JMIR Rehabil Assist Technol. 2026 Jan 7;13:e68286. doi: 10.2196/68286 (PMC12779105; doi:10.2196/68286)
Supplement: Multimedia Appendix 1 [file rehab-v13-e68286-s001.docx]

**Screenshot of the Hope Programme**

**
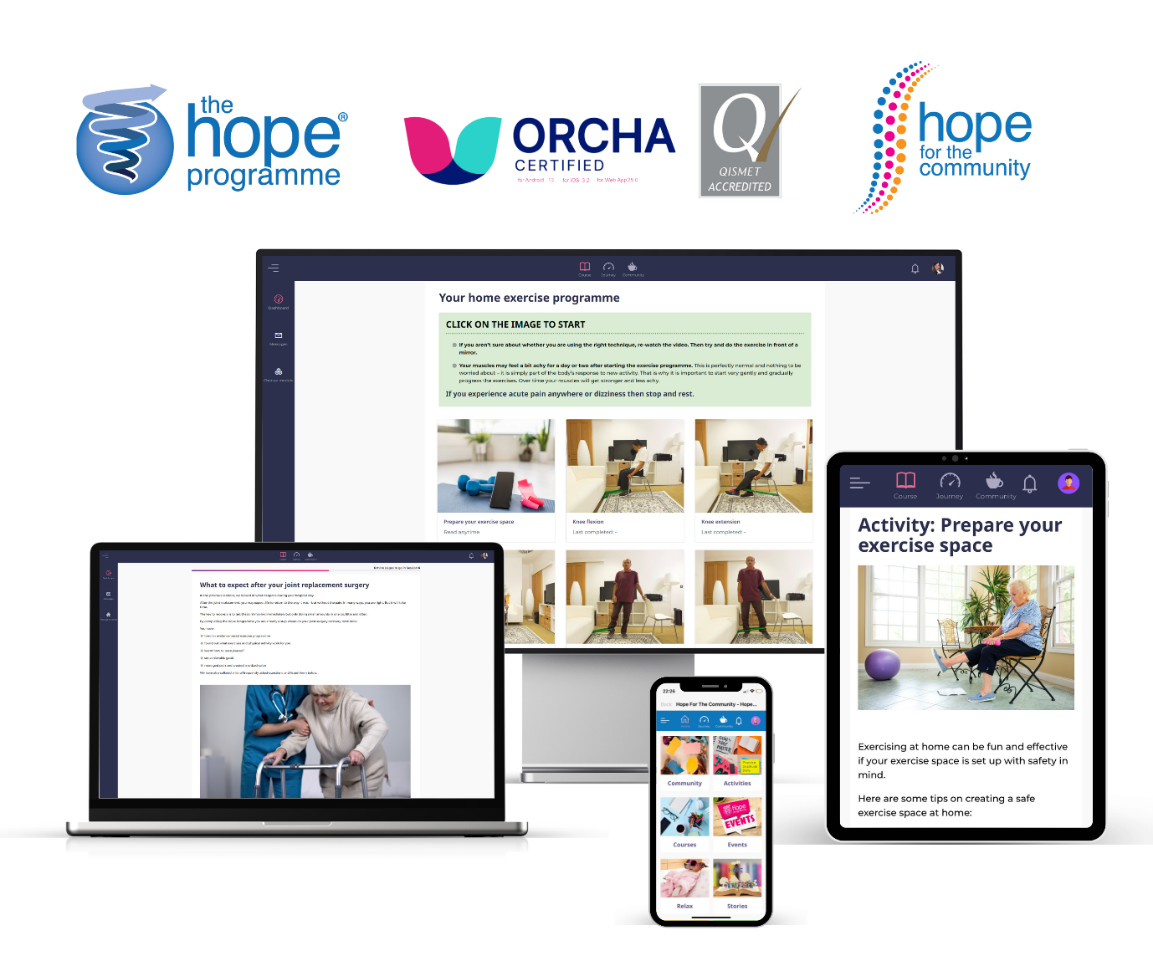
**

**Session 1: Instilling Hope**

Self-management for before, during and after hip/knee replacement. Getting used to the digital platform. Hope philosophy including positivity, gratitude, and self-compassion. Introduction to goal setting and tailored home exercise programme.

**Session 2: Managing pain and fatigue**

Movement quality and physical activity, understanding barriers to exercise, strategies for coping with pain and fatigue, and learning to pace activities to prevent the boom and bust cycle.

**Session 3: Stress and shifting your thinking**

Stress signs and learning coping tips including exercise and acceptance.

**Session 4: Communication**

Effective communication for getting the best support from family, peers, employers and health professionals.

**Session 5: Sleep and mindfulness**

Causes of sleep problems and practical sleep tips. Using mindfulness to enjoy the present and to stop worrying about the future.

**Session 6: Setbacks and hospital stay**

Tips for managing exercise and other setbacks including support from family and health professionals. Creating your setback plan. Preparing for surgery, including what happens at your pre-op assessment, getting ready for hospital and what to expect after surgery. Eating for strength and wellbeing.

**Session 7: Happiness and strengths**

Addressing emotional and physical changes post-surgery, focusing on body image and intimacy. Exploring how your strengths and your relationships can help adapt to these changes.

**Session 8: Moving on with Hope**

Tips for becoming more hopeful and happier, including finding your purpose and doing something pleasant.
