# Supplementary material for: Using a Co-Designed Digital Self-Management Program to Prepare Patients for Hip or Knee Replacement Surgery: Pragmatic Pilot Study
Source: JMIR Rehabil Assist Technol. 2026 Jan 7;13:e68286. doi: 10.2196/68286 (PMC12779105; doi:10.2196/68286)
Supplement: Multimedia Appendix 2 [file rehab-v13-e68286-s002.docx]

**Appendix for health economic evaluation analysis:**

**Appendix1: Parameter inputs**

Data parameters from the study (following data parameters were provided as outcomes from the study)

Number of interactions per patient (Mean number of interactions per patient - from Hope statistical analysis part)

| Type of consultation | Health / Social Care Professional | Baseline | | @ 8 weeks | | @ 6 months | |
| --- | --- | --- | --- | --- | --- | --- | --- |
|  |  | Mean | Std. error | Mean | Std. error | Mean | Std. error |
| Remote | Physiotherapist | 0 | NA | 0 | NA | 0 | NA |
| F2F | Physiotherapist | 0.29 | 0.16 | 0.07 | 0.07 | 0.43 | 0.43 |
| Remote | GP | 0.21 | 0.11 | 0.71 | 0.32 | 0.21 | 0.11 |
| F2F | GP | 0.64 | 0.25 | 0.36 | 0.17 | 0.21 | 0.11 |
| F2F | Home or Social Carer | 0 | NA | 0 | NA | 0 | NA |
| Remote | PALS or 111 call | 0 | NA | 0 | NA | 0 | NA |
| F2F | Visit to A&E | 0 | NA | 0 | NA | 0 | NA |
| F2F | Hospital visit | 0.14 | 0.1 | 0.21 | 0.11 | 0.29 | 0.19 |
| F2F | Ambulance call-out | 0 | NA | 0 | NA | 0 | NA |

**Seniority & Banding of Health & Social Care Professionals**

| Grouping | Health / Social Care Professional | Baseline | | @ 8 weeks | | @ 6 months | |
| --- | --- | --- | --- | --- | --- | --- | --- |
|  |  | % of total  visits | N visits | % of total  visits | N visits | % of total  visits | N visits |
| GP visits | Nurse-led primary care^i^ | 8.3% | 12 | 13.3% | 15 | 16.7% | 6 |
|  | GP | 83% |  | 86.7% |  | 66.7% |  |
|  | Phlebotomist | 8.3% |  | 0 |  | 0 |  |
|  | Pharmacist | 0 |  | 0 |  | 0 |  |
|  | Dietician | 0 |  | 0 |  | 16.7% |  |
| Hospital visits | Surgeon | 0 | 2 | 33.3% | 3 | 0 | 4 |
|  | Doctor^ii^ | 0 |  | 33.3% |  | 100% |  |
|  | Radiologist | 50% |  | 33.3% |  | 0 |  |
|  | Nurses | 50% |  | 0 |  | 0 |  |
| Physiotherapists | Senior (Band 8) | 7.1% | 4 | 7.1% | 4 | 7.1% | 4 |
|  | Physiotherapist (Band 7) | 39.3% |  | 39.3% |  | 39.3% |  |
|  | Physiotherapist (Band 6) | 39.3% |  | 39.3% |  | 39.3% |  |
|  | Research or Student (Band 5) | 14.3% |  | 14.3% |  | 14.3% |  |

### **Duration per interaction**

The mean duration per patient interaction was derived from NHS data tables and the published literature.

| Type of  consultation | Health / Social Care Professional | Mean duration  per interaction (minutes) | Std. Error | Source |
| --- | --- | --- | --- | --- |
| Remote | Physiotherapist | 40 | Generic Std. Error of 20% | [7] |
| F2F | Physiotherapist | 60 |  |  |
| Remote | GP | 3.2 |  | [4] |
| F2F | GP | 9.22 |  |  |
| F2F | Diabetes Nurse (primary care) | 12.8 |  | [8] |
| F2F | Primary Care Nurse (incl. Phlebotomy) | 7.5 |  | [9] |
| F2F | Hospital Nurse | 30 |  | [10] |
| F2F | Surgeon | 30 |  |  |
| F2F | Radiologist | 30 |  |  |
| F2F | Consultant | 30 |  |  |
| F2F | Registrar | 30 |  |  |

| Community | Band 7 | Physiotherapist | £ 1.13 |  |  |
| --- | --- | --- | --- | --- | --- |
| Community | Band 8a | Physiotherapist | £ 1.29 |  |  |
| Primary Care |  | GP | £ 4.64 | (incl. qualification costs)  including direct care staff costs |  |
| Primary Care |  | Primary Care  Nurse | £0.89 | (incl. qualification costs) same  as primary care Phlebotomist |  |
| Primary Care |  | Diabetes Nurse | £ 0.89 | (incl. qualification costs) |  |
| Primary Care | Band 6 | Pharmacist | £ 0.94 |  |  |
| Community/  Primary Care |  | Home or Social  Carer | £ 0.17 |  | [12] |
| Secondary Care | Bands 4-8a | Nurse | £ 0.89 (mean)  [£1.24 – £0.57] |  | [4] |
| Secondary Care | Consultant  - Surgical | Surgeon | £ 2.44 |  |  |
| Secondary Care | Associate  Specialist | Radiologist | £ 2.35 |  |  |
| Secondary Care | Consultant  - Medical | Consultant | £ 2.45 |  |  |
| Secondary Care |  | Registrar | £ 1.25 |  |  |

| Setting | NHS Band | Health / Social Care  Professional | Cost per minute  (GBP) - 2023 | Comment | Source |
| --- | --- | --- | --- | --- | --- |
| Hospital | Band 5 | Physiotherapist | £ 0.71 |  | [4] |
| Hospital | Band 6 | Physiotherapist | £ 0.91 |  |  |
| Hospital | Band 7 | Physiotherapist | £ 0.91 |  |  |
| Hospital | Band 8a | Physiotherapist | £ 1.25 |  |  |
| Community | Band 5 | Physiotherapist | £ 0.72 |  |  |
| Community | Band 6 | Physiotherapist | £ 0.94 |  |  |

*Other costs (Health & Social care cost per item (2023))*

| Cost item | Cost per item (GBP) - 2023 | | Source | |
| --- | --- | --- | --- | --- |
|  | Mean | Std. Error | Source | Comment |
| Visit to A&E | £ 270 | 72.96 | [5] | VB01Z – VB09Z |
| Session with Dietician | £ 102.9 | Generic Std. Error | [4] |  |
| PALS or 111 call | £ 16.87 | 8.6 | [13] | Table 12.10  2012 data converted to 2023 cost by using Bank of England CPI (’12-  23) |
| Ambulance call-out | £ 276.17 | Generic Std. Error | [5] | AMB-2 – See and treat |

# **Assumptions**

The following assumptions were made to enable the construction of the cost-impact analysis model:

- The NHS banding of Physiotherapists seen by patients during the study period

remains constant

- Phlebotomists in primary care cost the same as primary care nurses
- Diabetes nurses cost the same as primary care nurses
- The Homecare Cost of Care Exercise 2022-2023 carried out by Bradford Council can be applied to the Southwest of England^12^
- Cost per PALS call is the same as cost per 111 call
- Remote consultation with a physiotherapist takes the same as a community visit without the travel time
- All entries of medical secretary, receptionist, and paramedic have been costed as nurse-led primary care
- Duration of consult with Diabetes nurse and Pharmacist is the same in F2F as in remote
- Duration of Phlebotomy services in GP practices in Manchester are transferrable to the Southwest of England^9^
- An outpatient appointment in secondary care with a nurse is the same duration as with a doctor in secondary care
- The average consultation duration at Ashford & St. Peter's Hospital Trust for outpatient consultations is transferrable to the Southwest^10^
- Consultations with secondary care professionals are all Face-to-Face
- The cost of "GP Visits" and the cost of "Hospital Visits" are composed of a number of different healthcare professionals that patients interact with. We have assumed that the same mix of HCPs who deliver face-to-face consultations also deliver remote consultations.
- Hospital doctors seen by patients in this study are Consultants and Registrars (at a 50/50 split)
- Survey respondents who have indicated "other hospital visits" in the survey are referring to consultations with doctors in secondary care
- 50% of Physiotherapist visits/consultations are based in the community and 50% in Hospitals
- Where no data was provided by respondents, it was assumed that the number of interactions was 0
- No discounting was applied to costs
