# Supplementary material for: Using a Co-Designed Digital Self-Management Program to Prepare Patients for Hip or Knee Replacement Surgery: Pragmatic Pilot Study
Source: JMIR Rehabil Assist Technol. 2026 Jan 7;13:e68286. doi: 10.2196/68286 (PMC12779105; doi:10.2196/68286)
Supplement: Multimedia Appendix 3 [file rehab-v13-e68286-s003.docx]

**System Usability Scale scores (n=16).**

| Statements | Strongly disagree/disagree, n (%) | Neutral, n (%) | Strongly agree/agree, n (%) |
| --- | --- | --- | --- |
| I think that I would like to use the platform frequently | 1(6) | 8 (50) | 7 (44) |
| I found the platform unnecessarily complex | 12 (75) | 2 (12) | 2 (12) |
| I thought the platform was easy to use | 2 (12) | 2 (12) | 12 (75) |
| I think that I would need technical assistance to be able to use the platform | 12 (75) | 4 (25) | 0 (0) |
| I found the various functions in the platform were well integrated | 2 (12) | 6 (38) | 8 (50) |
| I thought there was too much inconsistency in the platform | 8 (50) | 8 (50) | 0 (0) |
| I would imagine that most people would learn to use the platform very quickly | 1 (6) | 4 (25) | 11 (69) |
| I found the platform very cumbersome to use | 11 (69) | 3 (19) | 2 (12) |
| I felt very confident using the platform | 0 (0) | 4 (25) | 12 (75) |
| I needed to learn a lot of things before I could get going with the platform | 8 (50) | 6 (38) | 2 (12) |
