# Supplementary material for: Using a Co-Designed Digital Self-Management Program to Prepare Patients for Hip or Knee Replacement Surgery: Pragmatic Pilot Study
Source: JMIR Rehabil Assist Technol. 2026 Jan 7;13:e68286. doi: 10.2196/68286 (PMC12779105; doi:10.2196/68286)
Supplement: Multimedia Appendix 4 [file rehab-v13-e68286-s004.docx]

Changes in Arthritis Self-efficacy Scale (ASES) scores in those who had received surgery by 6 months (n=25) versus those who had not yet had surgery (n=14).

| Outcomes | | Baseline,  median (IQR) | 8 weeks,  median (IQR) | 6 months,  median (IQR) |
| --- | --- | --- | --- | --- |
| **Confidence to manage pain (1-10, ↑ = better)** | | | | |
|  | Had surgery (n=25) | 3.8 (2.7- 5.5) | 5.0 (3.7- 5.9) | 7.6 (5.3- 8.7) |
|  | Not had surgery (n=14) | 3.8 (1.8- 5.8) | 4.1 (3.0- 4.8) | 4.1 (2.8- 5.3) |
| **Confidence to manage other symptoms (1-10, ↑ = better)** | | | | |
|  | Had surgery (n=25) | 4.5 (2.6- 5.5) | 5.5 (4.4- 6.8) | 7.0 (4.8- 8.4) |
|  | Not had surgery (n=14) | 4.3 (2.3- 5.8) | 4.6 (3.6- 6.6) | 4.3 (3.8- 6.7) |
