# Supplementary material for: Using a Co-Designed Digital Self-Management Program to Prepare Patients for Hip or Knee Replacement Surgery: Pragmatic Pilot Study
Source: JMIR Rehabil Assist Technol. 2026 Jan 7;13:e68286. doi: 10.2196/68286 (PMC12779105; doi:10.2196/68286)
Supplement: Checklist 2 [file rehab-v13-e68286-s006.pdf]

CONSORT-EHEALTH (V 1.6.1) - Submission/Publication Form

The CONSORT-EHEALTH checklist is intended for authors of randomized trials evaluating web-based and Internet-based applications/interventions, including mobile interventions, electronic games (incl multiplayer games), social media, certain telehealth applications, and other interactive and/or networked electronic applications. Some of the items (e.g. all subitems under item 5 - description of the intervention) may also be applicable for other study designs.

The goal of the CONSORT EHEALTH checklist and guideline is to be
a) a guide for reporting for authors of RCTs,
b) to form a basis for appraisal of an ehealth trial (in terms of validity)

CONSORT-EHEALTH items/subitems are MANDATORY reporting items for studies published in the Journal of Medical Internet Research and other journals / scientific societies endorsing the checklist.

Items numbered 1., 2., 3., 4a., 4b etc are original CONSORT or CONSORT-NPT (non-pharmacologic treatment) items.
Items with Roman numerals (i., ii, iii, iv etc.) are CONSORT-EHEALTH extensions/ clarifications.

As the CONSORT-EHEALTH checklist is still considered in a formative stage, we would ask that you also RATE ON A SCALE OF 1-5 how important/useful you feel each item is FOR THE PURPOSE OF THE CHECKLIST and reporting guideline (optional).

Mandatory reporting items are marked with a red *.
In the textboxes, either copy & paste the relevant sections from your manuscript into this form - please include any quotes from your manuscript in QUOTATION MARKS,
or answer directly by providing additional information not in the manuscript, or elaborating on why the item was not relevant for this study.

YOUR ANSWERS WILL BE PUBLISHED AS A SUPPLEMENTARY FILE TO YOUR PUBLICATION IN JMIR AND ARE CONSIDERED PART OF YOUR PUBLICATION (IF ACCEPTED).
Please fill in these questions diligently. Information will not be copyedited, so please use proper spelling and grammar, use correct capitalization, and avoid abbreviations.

DO NOT FORGET TO SAVE AS PDF _AND_ CLICK THE SUBMIT BUTTON SO YOUR ANSWERS ARE IN OUR DATABASE !!!

Citation Suggestion (if you append the pdf as Appendix we suggest to cite this paper in the caption):
Eysenbach G, CONSORT-EHEALTH Group
CONSORT-EHEALTH: Improving and Standardizing Evaluation Reports of Web-based and Mobile Health Interventions

J Med Internet Res 2011;13(4):e126
URL: http://www.jmir.org/2011/4/e126/
doi: 10.2196/jmir.1923
PMID: 22209829

apx242@gmail.com Switch accounts

Draft not saved

Not shared

* Indicates required question

Your name *

First Last

Elizabeth Horton

Primary Affiliation (short), City, Country *

University of Toronto, Toronto, Canada

Coventry University, Coventry, United Kingdom

Your e-mail address *

abc@gmail.com

apx242@coventry.ac.uk

Title of your manuscript *

Provide the (draft) title of your manuscript.

Does a Digital Self-Management Programme Prepare Patients for Hip or Knee Replacement Surgery: A Pragmatic Co-Designed Pilot Study

Name of your App/Software/Intervention *

If there is a short and a long/alternate name, write the short name first and add the long name in brackets.

The HOPE (Help Overcome Problems Effectiv

Evaluated Version (if any)

e.g. "V1", "Release 2017-03-01", "Version 2.0.27913"

Your answer

Language(s) *

What language is the intervention/app in? If multiple languages are available, separate by comma (e.g. "English, French")

English

URL of your Intervention Website or App

e.g. a direct link to the mobile app on app in appstore (itunes, Google Play), or URL of the website. If the intervention is a DVD or hardware, you can also link to an Amazon page.

https://www.h4c.org.uk

URL of an image/screenshot (optional)

Your answer

Accessibility *

Can an enduser access the intervention presently?

- ○ access is free and open
- ◉ access only for special usergroups, not open
- ○ access is open to everyone, but requires payment/subscription/in-app purchases
- ○ app/intervention no longer accessible
- ○ Other:

Primary Medical Indication/Disease/Condition *

e.g. "Stress", "Diabetes", or define the target group in brackets after the condition, e.g. "Autism (Parents of children with)", "Alzheimers (Informal Caregivers of)"

musculoskeletal conditions (awaiting surgery

Primary Outcomes measured in trial *

comma-separated list of primary outcomes reported in the trial

Patient preparedness for surgery

Secondary/other outcomes

Are there any other outcomes the intervention is expected to affect?

Health-related quality of life, symptoms, physical function, self-efficacy, pain, physical activity, usability, user engagement, resource utilization, user feedback

Recommended "Dose" *

What do the instructions for users say on how often the app should be used?

- ○ Approximately Daily
- ○ Approximately Weekly
- ○ Approximately Monthly
- ○ Approximately Yearly
- ◉ "as needed"
- ○ Other:

Approx. Percentage of Users (starters) still using the app as recommended after 3 months *

- ○ unknown / not evaluated
- ○ 0-10%
- ○ 11-20%
- ○ 21-30%
- ○ 31-40%
- ○ 41-50%
- ○ 51-60%
- ○ 61-70%
- ○ 71%-80%
- ○ 81-90%
- ○ 91-100%
- ◉ Other: 8 week intervention - 3 month users unknown

Overall, was the app/intervention effective? *

- ○ yes: all primary outcomes were significantly better in intervention group vs control
- ○ partly: SOME primary outcomes were significantly better in intervention group vs control
- ○ no statistically significant difference between control and intervention
- ○ potentially harmful: control was significantly better than intervention in one or more outcomes
- ○ inconclusive: more research is needed
- ◉ Other: The study was not powered to detect efficacy, only to test feasibility

Article Preparation Status/Stage *

At which stage in your article preparation are you currently (at the time you fill in this form)

- ○ not submitted yet - in early draft status
- ○ not submitted yet - in late draft status, just before submission
- ○ submitted to a journal but not reviewed yet
- ◉ submitted to a journal and after receiving initial reviewer comments
- ○ submitted to a journal and accepted, but not published yet
- ○ published
- ○ Other:

Journal *

If you already know where you will submit this paper (or if it is already submitted), please provide the journal name (if it is not JMIR, provide the journal name under "other")

- ○ not submitted yet / unclear where I will submit this
- ◉ Journal of Medical Internet Research (JMIR)
- ○ JMIR mHealth and UHealth
- ○ JMIR Serious Games
- ○ JMIR Mental Health
- ○ JMIR Public Health
- ○ JMIR Formative Research
- ○ Other JMIR sister journal
- ○ Other:

Is this a full powered effectiveness trial or a pilot/feasibility trial? *

- ◉ Pilot/feasibility
- ○ Fully powered

Manuscript tracking number *

If this is a JMIR submission, please provide the manuscript tracking number under "other" (The ms tracking number can be found in the submission acknowledgement email, or when you login as author in JMIR. If the paper is already published in JMIR, then the ms tracking number is the four-digit number at the end of the DOI, to be found at the bottom of each published article in JMIR)

- ○ no ms number (yet) / not (yet) submitted to / published in JMIR
- ◉ Other: #68286

TITLE AND ABSTRACT

1a) TITLE: Identification as a randomized trial in the title

1a) Does your paper address CONSORT item 1a? *

I.e does the title contain the phrase "Randomized Controlled Trial"? (if not, explain the reason under "other")

- ○ yes
- ◉ Other: As it was pilot study assessing the feasibility of the intervention no c

1a-i) Identify the mode of delivery in the title

Identify the mode of delivery. Preferably use “web-based” and/or “mobile” and/or “electronic game” in the title. Avoid ambiguous terms like “online”, “virtual”, “interactive”. Use “Internet-based” only if Intervention includes non-web-based Internet components (e.g. email), use “computer-based” or “electronic” only if offline products are used. Use “virtual” only in the context of “virtual reality” (3-D worlds). Use “online” only in the context of “online support groups”. Complement or substitute product names with broader terms for the class of products (such as “mobile” or “smart phone” instead of “iphone”), especially if the application runs on different platforms.

	1	2	3	4	5	
subitem not at all important	◉	○	○	○	○	essential

Clear selection

Does your paper address subitem 1a-i? *

Copy and paste relevant sections from manuscript title (include quotes in quotation marks "like this" to indicate direct quotes from your manuscript), or elaborate on this item by providing additional information not in the ms, or briefly explain why the item is not applicable/relevant for your study

We used the term "digital" to describe our program, which can be accessed via any internet enabled device e.g. smartphone, tablet, laptop

1a-ii) Non-web-based components or important co-interventions in title

Mention non-web-based components or important co-interventions in title, if any (e.g., “with telephone support”).

	1	2	3	4	5	
subitem not at all important	(●)	○	○	○	○	essential

Clear selection

Does your paper address subitem 1a-ii?

Copy and paste relevant sections from manuscript title (include quotes in quotation marks "like this" to indicate direct quotes from your manuscript), or elaborate on this item by providing additional information not in the ms, or briefly explain why the item is not applicable/relevant for your study

There are no non web based components to the program

1a-iii) Primary condition or target group in the title

Mention primary condition or target group in the title, if any (e.g., “for children with Type I Diabetes”) Example: A Web-based and Mobile Intervention with Telephone Support for Children with Type I Diabetes: Randomized Controlled Trial

	1	2	3	4	5	
subitem not at all important	○	○	○	○	◉	essential

Clear selection

Does your paper address subitem 1a-iii? *

Copy and paste relevant sections from manuscript title (include quotes in quotation marks "like this" to indicate direct quotes from your manuscript), or elaborate on this item by providing additional information not in the ms, or briefly explain why the item is not applicable/relevant for your study

"Does a Digital Self-Management Programme Prepare Patients for Hip or Knee Replacement Surgery: A Pragmatic Co-Designed Pilot Study"

1b) ABSTRACT: Structured summary of trial design, methods, results, and conclusions

NPT extension: Description of experimental treatment, comparator, care providers, centers, and blinding status.

1b-i) Key features/functionalities/components of the intervention and comparator in the METHODS section of the ABSTRACT

Mention key features/functionalities/components of the intervention and comparator in the abstract. If possible, also mention theories and principles used for designing the site. Keep in mind the needs of systematic reviewers and indexers by including important synonyms. (Note: Only report in the abstract what the main paper is reporting. If this information is missing from the main body of text, consider adding it)

	1	2	3	4	5	
subitem not at all important	○	◉	○	○	○	essential

Clear selection

Does your paper address subitem 1b-i? *

Copy and paste relevant sections from the manuscript abstract (include quotes in quotation marks "like this" to indicate direct quotes from your manuscript), or elaborate on this item by providing additional information not in the ms, or briefly explain why the item is not applicable/relevant for your study

The study employed a pragmatic, pre-post with follow-up single-arm design. All intervention and data collection components were delivered online. Following iterative co-development of the intervention, the content was refined and optimised into a final version for testing. The resulting programme was an eight-week intervention delivered via the Hope 4 The Community Interest Company (H4C) digital platform (Powered by H4C®). Data were collected at baseline (pre Hope Programme), 8 weeks (post Hope Programme) and 6 months follow-up.

1b-ii) Level of human involvement in the METHODS section of the ABSTRACT

Clarify the level of human involvement in the abstract, e.g., use phrases like “fully automated” vs. “therapist/nurse/care provider/physician-assisted” (mention number and expertise of providers involved, if any). (Note: Only report in the abstract what the main paper is reporting. If this information is missing from the main body of text, consider adding it)

	1	2	3	4	5	
subitem not at all important	(●)	○	○	○	○	essential

Clear selection

Does your paper address subitem 1b-ii?

Copy and paste relevant sections from the manuscript abstract (include quotes in quotation marks "like this" to indicate direct quotes from your manuscript), or elaborate on this item by providing additional information not in the ms, or briefly explain why the item is not applicable/relevant for your study

Participants were supported by a peer facilitator throughout the program. Peer facilitators respond to queries, stimulate conversations and encourage participants to support each other, via the in-program discussion forums, private messaging, and feedback functions.

1b-iii) Open vs. closed, web-based (self-assessment) vs. face-to-face assessments in the METHODS section of the ABSTRACT

Mention how participants were recruited (online vs. offline), e.g., from an open access website or from a clinic or a closed online user group (closed usergroup trial), and clarify if this was a purely web-based trial, or there were face-to-face components (as part of the intervention or for assessment). Clearly say if outcomes were self-assessed through questionnaires (as common in web-based trials). Note: In traditional offline trials, an open trial (open-label trial) is a type of clinical trial in which both the researchers and participants know which treatment is being administered. To avoid confusion, use "blinded" or "unblinded" to indicated the level of blinding instead of "open", as "open" in web-based trials usually refers to "open access" (i.e. participants can self-enrol). (Note: Only report in the abstract what the main paper is reporting. If this information is missing from the main body of text, consider adding it)

	1	2	3	4	5	
subitem not at all important	○	◉	○	○	○	essential

Clear selection

Does your paper address subitem 1b-iii?

Copy and paste relevant sections from the manuscript abstract (include quotes in quotation marks "like this" to indicate direct quotes from your manuscript), or elaborate on this item by providing additional information not in the ms, or briefly explain why the item is not applicable/relevant for your study

"Patients were recruited from those on the waiting list for hip or knee surgery"

1b-iv) RESULTS section in abstract must contain use data

Report number of participants enrolled/assessed in each group, the use/uptake of the intervention (e.g., attrition/adherence metrics, use over time, number of logins etc.), in addition to primary/secondary outcomes. (Note: Only report in the abstract what the main paper is reporting. If this information is missing from the main body of text, consider adding it)

	1	2	3	4	5	
subitem not at all important	○	○	○	○	◉	essential

Clear selection

Does your paper address subitem 1b-iv?

Copy and paste relevant sections from the manuscript abstract (include quotes in quotation marks "like this" to indicate direct quotes from your manuscript), or elaborate on this item by providing additional information not in the ms, or briefly explain why the item is not applicable/relevant for your study

Thirty-nine participants completed ≥5 of the 8 sessions and all surveys. Among the 25 participants who had surgery at 6 months, 92% felt better prepared due to the Hope Programme. Median improvements in most outcomes were observed at 8 weeks, with several continuing to improve at 6 months. The Friedman test showed significant improvements over 6 months in self-efficacy (pain: P=.002, other symptoms: P<.001), pain (P=.037), health status (P=.022), and mental wellbeing (P=.010). No significant changes were noted in physical activity. While the early cost analysis did not reach statistical significance, it indicated potential cost savings from reduced patient interactions with healthcare professionals. Sixty four percent (25/39) of participants had surgery and this likely contributed in part to improvements in outcomes. System usability was rated above average (mean score 70.1), and post-programme feedback was generally positive, highlighting peer support, coping strategies, and better preparation for surgery.

1b-v) CONCLUSIONS/DISCUSSION in abstract for negative trials

Conclusions/Discussions in abstract for negative trials: Discuss the primary outcome - if the trial is negative (primary outcome not changed), and the intervention was not used, discuss whether negative results are attributable to lack of uptake and discuss reasons. (Note: Only report in the abstract what the main paper is reporting. If this information is missing from the main body of text, consider adding it)

	1	2	3	4	5	
subitem not at all important	(•)	( )	( )	( )	( )	essential

Clear selection

Does your paper address subitem 1b-v?

Copy and paste relevant sections from the manuscript abstract (include quotes in quotation marks "like this" to indicate direct quotes from your manuscript), or elaborate on this item by providing additional information not in the ms, or briefly explain why the item is not applicable/relevant for your study

The results are promising in relation to participants attending the Hope Programme feeling better prepared for surgery.

INTRODUCTION

2a) In INTRODUCTION: Scientific background and explanation of rationale

2a-i) Problem and the type of system/solution

Describe the problem and the type of system/solution that is object of the study: intended as stand-alone intervention vs. incorporated in broader health care program? Intended for a particular patient population? Goals of the intervention, e.g., being more cost-effective to other interventions, replace or complement other solutions? (Note: Details about the intervention are provided in “Methods” under 5)

	1	2	3	4	5	
subitem not at all important	○	○	○	◉	○	essential

Clear selection

Does your paper address subitem 2a-i? *

Copy and paste relevant sections from the manuscript (include quotes in quotation marks "like this" to indicate direct quotes from your manuscript), or elaborate on this item by providing additional information not in the ms, or briefly explain why the item is not applicable/relevant for your study

"The aim of this pragmatic, multi methods study was to optimise and evaluate the Hope Programme to determine whether patients were better prepared for surgery. The study objectives included optimising the Hope Programme through co-design with stakeholders, implementing and testing the programme with patients waiting for a joint replacement, and assessing their preparedness for surgery."

2a-ii) Scientific background, rationale: What is known about the (type of) system

Scientific background, rationale: What is known about the (type of) system that is the object of the study (be sure to discuss the use of similar systems for other conditions/ diagnoses, if appropiate), motivation for the study, i.e. what are the reasons for and what is the context for this specific study, from which stakeholder viewpoint is the study performed, potential impact of findings [2]. Briefly justify the choice of the comparator.

	1	2	3	4	5	
subitem not at all important	○	○	◉	○	○	essential

Clear selection

Does your paper address subitem 2a-ii? *

Copy and paste relevant sections from the manuscript (include quotes in quotation marks "like this" to indicate direct quotes from your manuscript), or elaborate on this item by providing additional information not in the ms, or briefly explain why the item is not applicable/relevant for your study

"In a review [17] of over 30 prehabilitation surgery schools in the UK and Ireland (these schools inform patients about what to expect and guide them on how to prepare physically and mentally to reduce postoperative risks of surgery) only 40% contained content to manage emotional wellbeing and only 13% used digital apps. Further, many interventions were not underpinned by behaviour change theory and techniques."

2b) In INTRODUCTION: Specific objectives or hypotheses

Does your paper address CONSORT subitem 2b? *

Copy and paste relevant sections from the manuscript (include quotes in quotation marks "like this" to indicate direct quotes from your manuscript), or elaborate on this item by providing additional information not in the ms, or briefly explain why the item is not applicable/relevant for your study

The specific objectives were related to the feasibility of the intervention

METHODS

3a) Description of trial design (such as parallel, factorial) including allocation ratio

Does your paper address CONSORT subitem 3a? *

Copy and paste relevant sections from the manuscript (include quotes in quotation marks "like this" to indicate direct quotes from your manuscript), or elaborate on this item by providing additional information not in the ms, or briefly explain why the item is not applicable/relevant for your study

"This study employed a co-design phase followed by a pragmatic, pre-post, with follow-up, single arm intervention study."

3b) Important changes to methods after trial commencement (such as eligibility criteria), with reasons

Does your paper address CONSORT subitem 3b? *

Copy and paste relevant sections from the manuscript (include quotes in quotation marks "like this" to indicate direct quotes from your manuscript), or elaborate on this item by providing additional information not in the ms, or briefly explain why the item is not applicable/relevant for your study

No changes were made to the methods after trial commencement

3b-i) Bug fixes, Downtimes, Content Changes

Bug fixes, Downtimes, Content Changes: ehealth systems are often dynamic systems. A description of changes to methods therefore also includes important changes made on the intervention or comparator during the trial (e.g., major bug fixes or changes in the functionality or content) (5-iii) and other “unexpected events” that may have influenced study design such as staff changes, system failures/downtimes, etc. [2].

	1	2	3	4	5	
subitem not at all important	○	○	○	◉	○	essential

Clear selection

Does your paper address subitem 3b-i?

Copy and paste relevant sections from the manuscript (include quotes in quotation marks "like this" to indicate direct quotes from your manuscript), or elaborate on this item by providing additional information not in the ms, or briefly explain why the item is not applicable/relevant for your study

The study was not affected by any of the above issues

4a) Eligibility criteria for participants

Does your paper address CONSORT subitem 4a? *

Copy and paste relevant sections from the manuscript (include quotes in quotation marks "like this" to indicate direct quotes from your manuscript), or elaborate on this item by providing additional information not in the ms, or briefly explain why the item is not applicable/relevant for your study

"Broad eligibility criteria were applied, to maximise inclusivity and opportunity to take part. Inclusion criteria were as follows:

- Adult (18 years or over)
- Located in the United Kingdom (South West of England)
- On a waiting list for hip and knee replacement surgery
- Access to the internet and a device allowing them to engage with the intervention
- Be able to engage with all the material in the intervention."

4a-i) Computer / Internet literacy

Computer / Internet literacy is often an implicit "de facto" eligibility criterion - this should be explicitly clarified.

	1	2	3	4	5	
subitem not at all important	○	○	○	◉	○	essential

Clear selection

Does your paper address subitem 4a-i?

Copy and paste relevant sections from the manuscript (include quotes in quotation marks "like this" to indicate direct quotes from your manuscript), or elaborate on this item by providing additional information not in the ms, or briefly explain why the item is not applicable/relevant for your study

"• Access to the internet and a device allowing them to engage with the intervention
• Be able to engage with all the material in the intervention."

4a-ii) Open vs. closed, web-based vs. face-to-face assessments:

Open vs. closed, web-based vs. face-to-face assessments: Mention how participants were recruited (online vs. offline), e.g., from an open access website or from a clinic, and clarify if this was a purely web-based trial, or there were face-to-face components (as part of the intervention or for assessment), i.e., to what degree got the study team to know the participant. In online-only trials, clarify if participants were quasi-anonymous and whether having multiple identities was possible or whether technical or logistical measures (e.g., cookies, email confirmation, phone calls) were used to detect/prevent these.

	1	2	3	4	5	
subitem not at all important	○	○	○	◉	○	essential

Clear selection

Does your paper address subitem 4a-ii? *

Copy and paste relevant sections from the manuscript (include quotes in quotation marks "like this" to indicate direct quotes from your manuscript), or elaborate on this item by providing additional information not in the ms, or briefly explain why the item is not applicable/relevant for your study

"Patients interested in attending the Hope Programme were referred to the study sign-up webpage through several routes. NHS South West referral sources included secondary care, primary care, and MSK clinics. Eligible participants were referred directly to H4C to enrol on the Hope Programme and given the option to take part in the research study. Patients who chose to take part in the study were directed to the Participant Information Sheet and consent form in Qualtrics Survey Software. Patients were made aware that there was no obligation to take part in the study, and that their decision would not affect their quality of care."

4a-iii) Information giving during recruitment

Information given during recruitment. Specify how participants were briefed for recruitment and in the informed consent procedures (e.g., publish the informed consent documentation as appendix, see also item X26), as this information may have an effect on user self-selection, user expectation and may also bias results.

	1	2	3	4	5	
subitem not at all important	○	○	○	○	◉	essential

Clear selection

Does your paper address subitem 4a-iii?

Copy and paste relevant sections from the manuscript (include quotes in quotation marks "like this" to indicate direct quotes from your manuscript), or elaborate on this item by providing additional information not in the ms, or briefly explain why the item is not applicable/relevant for your study

available on request. "Patients who chose to take part in the study were directed to the Participant Information Sheet and consent form in Qualtrics Survey Software."

4b) Settings and locations where the data were collected

Does your paper address CONSORT subitem 4b? *

Copy and paste relevant sections from the manuscript (include quotes in quotation marks "like this" to indicate direct quotes from your manuscript), or elaborate on this item by providing additional information not in the ms, or briefly explain why the item is not applicable/relevant for your study

"The intervention and data collection took place online."

4b-i) Report if outcomes were (self-)assessed through online questionnaires

Clearly report if outcomes were (self-)assessed through online questionnaires (as common in web-based trials) or otherwise.

	1	2	3	4	5	
subitem not at all important	○	○	○	◉	○	essential

Clear selection

Does your paper address subitem 4b-i? *

Copy and paste relevant sections from the manuscript (include quotes in quotation marks "like this" to indicate direct quotes from your manuscript), or elaborate on this item by providing additional information not in the ms, or briefly explain why the item is not applicable/relevant for your study

All study data were collected online via questionnaires administered through Qualtrics Survey Software (Qualtrics 2019, Provo, UT, USA, Available from: http://

4b-ii) Report how institutional affiliations are displayed

Report how institutional affiliations are displayed to potential participants [on ehealth media], as affiliations with prestigious hospitals or universities may affect volunteer rates, use, and reactions with regards to an intervention.(Not a required item – describe only if this may bias results)

	1	2	3	4	5	
subitem not at all important	○	○	◉	○	○	essential

Clear selection

Does your paper address subitem 4b-ii?

Copy and paste relevant sections from the manuscript (include quotes in quotation marks "like this" to indicate direct quotes from your manuscript), or elaborate on this item by providing additional information not in the ms, or briefly explain why the item is not applicable/relevant for your study

The Coventry University logos were displayed on study banner across online surveys

5) The interventions for each group with sufficient details to allow replication, including how and when they were actually administered

5-i) Mention names, credential, affiliations of the developers, sponsors, and owners

Mention names, credential, affiliations of the developers, sponsors, and owners [6] (if authors/evaluators are owners or developer of the software, this needs to be declared in a “Conflict of interest” section or mentioned elsewhere in the manuscript).

	1	2	3	4	5	
subitem not at all important	○	○	◉	○	○	essential

Clear selection

Does your paper address subitem 5-i?

Copy and paste relevant sections from the manuscript (include quotes in quotation marks "like this" to indicate direct quotes from your manuscript), or elaborate on this item by providing additional information not in the ms, or briefly explain why the item is not applicable/relevant for your study

The digital platform, called Powered by H4C®, is owned by Hope For The Community (H4C)
Community Interest Company, a small UK-based social enterprise. H4C is a spin-out company of Coventry University. Co-author Andy Turner is co-founder of the HOPE

5-ii) Describe the history/development process

Describe the history/development process of the application and previous formative evaluations (e.g., focus groups, usability testing), as these will have an impact on adoption/use rates and help with interpreting results.

	1	2	3	4	5	
subitem not at all important	○	○	○	◉	○	essential

Clear selection

Does your paper address subitem 5-ii?

Copy and paste relevant sections from the manuscript (include quotes in quotation marks "like this" to indicate direct quotes from your manuscript), or elaborate on this item by providing additional information not in the ms, or briefly explain why the item is not applicable/relevant for your study

"In 2022, Coventry University and its University spin-out social enterprise, Hope 4 The Community interest company (H4C) developed a proof-of-concept digital intervention, called the Hope Programme, to help patients prepare for hip and knee surgery. The Hope Programme for hip and knee patients shares the same underlying theoretical framework as other Hope Programmes for long-term conditions offered by H4C, which have been taxonomized using the taxonomy of self-management support [18] and are described in detail in published articles [19–21]. All 14 digital versions of the Hope Programme have been approved by the Quality Institute for Self-Management Education and Training (QISMET) for the provision of self-management structured education (QIS2020 and QIS2023 [22]) and certified by the Organisation for the Review of Care and Health Apps (ORCHA [23]), the world's leading platform for digital health service reviews scoring 88% for Android and iOS, and 86% for WebApp. The high ratings indicate compliance with best practice in data and security, professional assurance and usability/accessibility.

The Hope Programme for hip and knee patients combines evidence-based self-management content with a validated exercise programme, incorporating a home exercise component tailored to individual needs and abilities, drawing from the work of Ageberg et al. [24].

In 2023 H4C were awarded funding through the UK Research and Innovation Healthy Ageing Challenge Scaling Social Ventures competition, to co-design and evaluate the Hope Programme for hip and knee surgery patients. The funding competition was to support social enterprises in scaling products and services to support healthy ageing and deliver social value."

5-iii) Revisions and updating

Revisions and updating. Clearly mention the date and/or version number of the application/intervention (and comparator, if applicable) evaluated, or describe whether the intervention underwent major changes during the evaluation process, or whether the development and/or content was “frozen” during the trial. Describe dynamic components such as news feeds or changing content which may have an impact on the replicability of the intervention (for unexpected events see item 3b).

	1	2	3	4	5	
subitem not at all important	○	○	○	●	○	essential

Clear selection

Does your paper address subitem 5-iii?

Copy and paste relevant sections from the manuscript (include quotes in quotation marks "like this" to indicate direct quotes from your manuscript), or elaborate on this item by providing additional information not in the ms, or briefly explain why the item is not applicable/relevant for your study

The content was frozen during the trial

5-iv) Quality assurance methods

Provide information on quality assurance methods to ensure accuracy and quality of information provided [1], if applicable.

	1	2	3	4	5	
subitem not at all important	○	○	○	●	○	essential

Clear selection

Does your paper address subitem 5-iv?

Copy and paste relevant sections from the manuscript (include quotes in quotation marks "like this" to indicate direct quotes from your manuscript), or elaborate on this item by providing additional information not in the ms, or briefly explain why the item is not applicable/relevant for your study

"Throughout the programme, participants were supported by two facilitators who were trained in line with QISMET standards. "

5-v) Ensure replicability by publishing the source code, and/or providing screenshots/screen-capture video, and/or providing flowcharts of the algorithms used

Ensure replicability by publishing the source code, and/or providing screenshots/screen-capture video, and/or providing flowcharts of the algorithms used. Replicability (i.e., other researchers should in principle be able to replicate the study) is a hallmark of scientific reporting.

	1	2	3	4	5	
subitem not at all important	○	○	○	◉	○	essential

Clear selection

Does your paper address subitem 5-v?

Copy and paste relevant sections from the manuscript (include quotes in quotation marks "like this" to indicate direct quotes from your manuscript), or elaborate on this item by providing additional information not in the ms, or briefly explain why the item is not applicable/relevant for your study

Screen shots are provided in the multimedia appendix 1, and we provide screenshots of the HOPE Program in our previous paper published in JMIR
which can be accessed here: https://www.jmir.org/2020/5/e17824/

5-vi) Digital preservation

Digital preservation: Provide the URL of the application, but as the intervention is likely to change or disappear over the course of the years; also make sure the intervention is archived (Internet Archive, webcitation.org, and/or publishing the source code or screenshots/videos alongside the article). As pages behind login screens cannot be archived, consider creating demo pages which are accessible without login.

	1	2	3	4	5	
subitem not at all important	○	◉	○	○	○	essential

Clear selection

Does your paper address subitem 5-vi?

Copy and paste relevant sections from the manuscript (include quotes in quotation marks "like this" to indicate direct quotes from your manuscript), or elaborate on this item by providing additional information not in the ms, or briefly explain why the item is not applicable/relevant for your study

"The programme content was organised into themed sessions across the 8 weeks as follows with an integrated tailored exercise programme
(described above) (See Multimedia Appendix 1 for brief description of each session content and screenshots of the intervention):

Session 1: Instilling Hope
Session 2: Managing pain and fatigue
Session 3: Stress and shifting your thinking
Session 4: Communication
Session 5: Sleep and mindfulness
Session 6: Setbacks and hospital stay
Session 7: Happiness and strengths
Session 8: Moving on with Hope"

H4C can provide demo pages for the HOPE Program on request. Please email hope@h4c.org.uk

5-vii) Access

Access: Describe how participants accessed the application, in what setting/context, if they had to pay (or were paid) or not, whether they had to be a member of specific group. If known, describe how participants obtained “access to the platform and Internet” [1]. To ensure access for editors/reviewers/readers, consider to provide a “backdoor” login account or demo mode for reviewers/readers to explore the application (also important for archiving purposes, see vi).

	1	2	3	4	5	
subitem not at all important	○	○	○	○	◉	essential

Clear selection

Does your paper address subitem 5-vii? *

Copy and paste relevant sections from the manuscript (include quotes in quotation marks "like this" to indicate direct quotes from your manuscript), or elaborate on this item by providing additional information not in the ms, or briefly explain why the item is not applicable/relevant for your study

"Patients interested in attending the Hope Programme were referred to the study sign-up webpage through several routes. NHS South West referral sources included secondary care, primary care, and MSK clinics. Eligible participants were referred directly to H4C to enrol on the Hope Programme and given the option to take part in the research study. Patients who chose to take part in the study were directed to the Participant Information Sheet and consent form in Qualtrics Survey Software."

5-viii) Mode of delivery, features/functionalities/components of the intervention and comparator, and the theoretical framework

Describe mode of delivery, features/functionalities/components of the intervention and comparator, and the theoretical framework [6] used to design them (instructional strategy [1], behaviour change techniques, persuasive features, etc., see e.g., [7, 8] for terminology). This includes an in-depth description of the content (including where it is coming from and who developed it) [1]," whether [and how] it is tailored to individual circumstances and allows users to track their progress and receive feedback" [6]. This also includes a description of communication delivery channels and – if computer-mediated communication is a component – whether communication was synchronous or asynchronous [6]. It also includes information on presentation strategies [1], including page design principles, average amount of text on pages, presence of hyperlinks to other resources, etc. [1].

	1	2	3	4	5	
subitem not at all important	◉	○	○	○	○	essential

Clear selection

Does your paper address subitem 5-viii? *

Copy and paste relevant sections from the manuscript (include quotes in quotation marks "like this" to indicate direct quotes from your manuscript), or elaborate on this item by providing additional information not in the ms, or briefly explain why the item is not applicable/relevant for your study

"The resulting programme is comprised of eight modules. and were hosted on H4C's digital platform, called Powered by H4C®. The content comprises text, images, videos, downloadable documents, and interactive activities (e.g. quizzes, self-monitoring tools, diaries) and discussion forums and messaging facilities. The digital content is released at set times over the 8 weeks but can be accessed at any time (asynchronous). Participants had the option to "fast track" the content if they were due to have surgery during the 8 week period.

Once accessed and viewed, the app content could be viewed offline, reducing the need for a data plan or high-quality internet connection.
"

5-ix) Describe use parameters

Describe use parameters (e.g., intended “doses” and optimal timing for use). Clarify what instructions or recommendations were given to the user, e.g., regarding timing, frequency, heaviness of use, if any, or was the intervention used ad libitum.

	1	2	3	4	5	
subitem not at all important	○	○	○	◉	○	essential

Clear selection

Does your paper address subitem 5-ix?

Copy and paste relevant sections from the manuscript (include quotes in quotation marks "like this" to indicate direct quotes from your manuscript), or elaborate on this item by providing additional information not in the ms, or briefly explain why the item is not applicable/relevant for your study

"Following completion of the pre Hope Programme survey, participants were given access to the Hope Programme (start date 13th or 27th July 2023) through a personalised log-in link.

Throughout the programme, participants were supported by two facilitators who were trained in line with QISMET standards. The programme content was organised into themed sessions across the 8 weeks as follows with an integrated tailored exercise programme (described above) (See Multimedia Appendix 1 for brief description of each session content and screenshots of the intervention):

Session 1: Instilling Hope
Session 2: Managing pain and fatigue
Session 3: Stress and shifting your thinking
Session 4: Communication
Session 5: Sleep and mindfulness
Session 6: Setbacks and hospital stay
Session 7: Happiness and strengths

5-x) Clarify the level of human involvement

Clarify the level of human involvement (care providers or health professionals, also technical assistance) in the e-intervention or as co-intervention (detail number and expertise of professionals involved, if any, as well as “type of assistance offered, the timing and frequency of the support, how it is initiated, and the medium by which the assistance is delivered”. It may be necessary to distinguish between the level of human involvement required for the trial, and the level of human involvement required for a routine application outside of a RCT setting (discuss under item 21 – generalizability).

	1	2	3	4	5	
subitem not at all important	○	○	◉	○	○	essential

Clear selection

Does your paper address subitem 5-x?

Copy and paste relevant sections from the manuscript (include quotes in quotation marks "like this" to indicate direct quotes from your manuscript), or elaborate on this item by providing additional information not in the ms, or briefly explain why the item is not applicable/relevant for your study

"Throughout the programme, participants were supported by two facilitators who were trained in line with QISMET standards"

5-xi) Report any prompts/reminders used

Report any prompts/reminders used: Clarify if there were prompts (letters, emails, phone calls, SMS) to use the application, what triggered them, frequency etc. It may be necessary to distinguish between the level of prompts/reminders required for the trial, and the level of prompts/reminders for a routine application outside of a RCT setting (discuss under item 21 – generalizability).

	1	2	3	4	5	
subitem not at all important	○	○	◉	○	○	essential

Clear selection

Does your paper address subitem 5-xi? *

Copy and paste relevant sections from the manuscript (include quotes in quotation marks "like this" to indicate direct quotes from your manuscript), or elaborate on this item by providing additional information not in the ms, or briefly explain why the item is not applicable/relevant for your study

Participants receive an email each week to inform them that new Program content is available. Automated email prompts are sent if participants have not logged in to the intervention for a period of time e.g. one week.

Participants are emailed a link to the study questionnaires (at T0, T1), and two automated reminders are sent via email if participants have not accessed the questionnaires e.g. after 7 days and after 14 days.

5-xii) Describe any co-interventions (incl. training/support)

Describe any co-interventions (incl. training/support): Clearly state any interventions that are provided in addition to the targeted eHealth intervention, as ehealth intervention may not be designed as stand-alone intervention. This includes training sessions and support [1]. It may be necessary to distinguish between the level of training required for the trial, and the level of training for a routine application outside of a RCT setting (discuss under item 21 – generalizability.

	1	2	3	4	5	
subitem not at all important	○	◉	○	○	○	essential

Clear selection

Does your paper address subitem 5-xii? *

Copy and paste relevant sections from the manuscript (include quotes in quotation marks "like this" to indicate direct quotes from your manuscript), or elaborate on this item by providing additional information not in the ms, or briefly explain why the item is not applicable/relevant for your study

Facilitators receive formal training and assessment (QISMET) in self management support and health coaching. Peer facilitators spend approximately 2 hours per week supporting participants on the online course.

6a) Completely defined pre-specified primary and secondary outcome measures, including how and when they were assessed

Does your paper address CONSORT subitem 6a? *

Copy and paste relevant sections from the manuscript (include quotes in quotation marks "like this" to indicate direct quotes from your manuscript), or elaborate on this item by providing additional information not in the ms, or briefly explain why the item is not applicable/relevant for your study

"Surgery preparation
At 6 months follow-up, participants were asked if they felt better prepared for surgery using the following question from the Patient Preparedness for Surgery questionnaire [33]: "Overall, I feel or felt (if had surgery) prepared for my upcoming surgery." There are six responses: strongly agree, agree, somewhat agree, somewhat disagree, disagree, strongly disagree. Participants were asked to provide reasons for their answers. Participants who had surgery also indicated which time point, before surgery, after surgery, or both before and after surgery they felt the Hope Programme helped them prepare. Participants provided textual responses to explain why they agreed or disagreed that the Hope Programme helped them prepare for surgery.

The following validated patient-reported outcome measures (PROMS) were collected at baseline, Hope Programme (8 weeks) and 6 months follow-up, via Qualtrics.

The Warwick Edinburgh Mental Wellbeing Scale – Short Version (SWEMWBS)
The SWEMWBS [34] is a short version assesses mental wellbeing within the adult population. The SWEMWBS uses seven items from the full WEMWBS [35] which relate more to mental functioning than feelings. The seven statements are positively worded with five response categories from 'none of the time' to 'all of the time'. Total scores range from 7 to 35, with higher scores indicating higher mental wellbeing. A change of one point of more on SWEMWBS total score represents a minimally important level of change.

The EuroQol index (EQ 5D index) & EuroQol Visual Analogue Scale (EQ VAS)
The EQ 5D index [36] and the EQ VAS are widely used measures of health status and health-related quality of life respectively). The EQ-5D index assesses patients' health state across five dimensions (self-care, mobility, anxiety/depression, usual activities and pain/discomfort) that are weighted to provide a utility value based on a population tariff. Scores range from 0 (death) to a 100 (perfect health). The EQ VAS is a vertical rating scale health scored between 0 (worst imaginable health) and 100 (best imaginable health).

Western Ontario and McMaster Universities Arthritis Index (WOMAC)
The Western Ontario and McMaster Universities Arthritis Index (WOMAC; [37]) consists of 24 items divided into 3 subscales: Pain (5 items); Stiffness (2 items); Physical Function (17 items). Items are scored on a scale of 0-4, which correspond to: None (0), Mild (1), Moderate (2), Severe (3), and Extreme (4). The scores for each subscale are summed up, with a possible score range of 0-20 for Pain, 0-8 for Stiffness, and 0-68 for Physical Function. A sum of the scores for all three subscales gives a total WOMAC score (maximum 96). Higher scores indicate worse pain, stiffness, and functional limitations.

Arthritis Self-Efficacy Scale (ASES)
The Arthritis Self-Efficacy Scale (ASES; [38]) measures a person's confidence to self-manage their arthritis symptoms and consists of two subscales: pain (5 items), and other symptoms (6 items). Items are scored 1 (very uncertain) to 10 (very certain). The scores for each subscale are summed, with a possible score range of 10-50 for pain and 10-60 for other symptoms. Higher scores indicate higher self-efficacy.

International Physical Activity Questionnaire short form (IPAQ-SF)
IPAQ-SF [39] assesses physical activity undertaken across a comprehensive set of domains including: a. leisure time physical activity; b. domestic and gardening (yard) activities; c. work-related physical activity; d. transport-related physical activity. The items are structured to provide separate scores on walking; moderate-intensity; and vigorous-

are structured to provide separate scores on walking, moderate intensity, and vigorous intensity activity as well as a combined total score to describe overall level of activity. Computation of the total score requires summation of the duration (in minutes) and frequency (days) of walking, moderate-intensity and vigorous-intensity activity. The IPAQ-SF scoring protocol assigns the following MET values to walking, moderate, and vigorous intensity activity: 3.3 METs, 4.0 METs, and 8.0 METs, respectively. Participants are considered to have met Centers for Disease Control & Prevention/American College of Sports Medicine (CDC/ACSM) physical activity recommendations if they reported at least 150 min/week of walking, moderate, or vigorous intensity physical activity.

Numerical Pain Rating Scale (NPRS)
The Numerical Rating Scale (NPRS-11; [40]) is an 11-point scale for self-report of pain. It is the most commonly used unidimensional pain scale. The respondent selects a whole number (integers 0–10) that best reflects the intensity (or other quality if requested of his/her pain. The anchors are 0 = no pain and 10 = worst possible pain (there are various different wordings of the upper anchor).

Hope Programme usability, user satisfaction and user engagement

Usability
The usability of the system was assessed by the System Usability Scale (SUS; [41], which was embedded into the last session of the Hope Programme. It was optional for participants to complete.   The SUS uses a 5-point Likert scale ranging from 1=strongly disagree to 5=strongly agree across 10 items. Odd numbered questions (1,3, 5, 7, 9) generate a positive response. Even numbered questions (2, 4, 6, 8, 10), generate a negative response which have to be inverted. All the points added up together could gain a maximum of forty, thus the multiplication by 2.5 to make the scale out of 100. A total score of ≥68 is considered above-average usability.

User engagement
The intervention platform collected user engagement data. For this study we report the number of sessions completed; the number of participants who used the personalised exercise programme; the most commonly bookmarked content/activities."

6a-i) Online questionnaires: describe if they were validated for online use and apply CHERRIES items to describe how the questionnaires were designed/deployed

If outcomes were obtained through online questionnaires, describe if they were validated for online use and apply CHERRIES items to describe how the questionnaires were designed/deployed [9].

	1	2	3	4	5	
subitem not at all important	○	○	◉	○	○	essential

Clear selection

Does your paper address subitem 6a-i?

Copy and paste relevant sections from manuscript text

CHERRIES checklist used - see multimedia appendix

6a-ii) Describe whether and how “use” (including intensity of use/dosage) was defined/measured/monitored

Describe whether and how “use” (including intensity of use/dosage) was defined/measured/monitored (logins, logfile analysis, etc.). Use/adoption metrics are important process outcomes that should be reported in any ehealth trial.

	1	2	3	4	5	
subitem not at all important	○	○	○	◉	○	essential

Clear selection

Does your paper address subitem 6a-ii?

Copy and paste relevant sections from manuscript text

"Given this was a feasibility study with complete case analysis as the pre-specified approach, we focused on participants who engaged with ≥5 sessions and completed follow-ups. This decision was made because: (1) the primary aim was assessing intervention feasibility/acceptability under optimal conditions; (2) minimal data was available from non-completers (only 4/15 provided follow-ups); and (3) high follow-up rates (98% at 8 weeks, 93% at 6 months) reduced concerns about attrition bias." The intervention platform collects user engagement data such as number of pages viewed in each session and pages that are bookmarked.

6a-iii) Describe whether, how, and when qualitative feedback from participants was obtained

Describe whether, how, and when qualitative feedback from participants was obtained (e.g., through emails, feedback forms, interviews, focus groups).

	1	2	3	4	5	
subitem not at all important	○	○	○	◉	○	essential

Clear selection

Does your paper address subitem 6a-iii?

Copy and paste relevant sections from manuscript text

Participants could provide free-text responses on the usability feedback questionnaire post-

6b) Any changes to trial outcomes after the trial commenced, with reasons

Does your paper address CONSORT subitem 6b? *

Copy and paste relevant sections from the manuscript (include quotes in quotation marks "like this" to indicate direct quotes from your manuscript), or elaborate on this item by providing additional information not in the ms, or briefly explain why the item is not applicable/relevant for your study

No changes were made to trial outcomes after the trial commenced.

7a) How sample size was determined

NPT: When applicable, details of whether and how the clustering by care provides or centers was addressed

7a-i) Describe whether and how expected attrition was taken into account when calculating the sample size

Describe whether and how expected attrition was taken into account when calculating the sample size.

	1	2	3	4	5	
subitem not at all important	○	○	◉	○	○	essential

Clear selection

Does your paper address subitem 7a-i?

Copy and paste relevant sections from manuscript title (include quotes in quotation marks "like this" to indicate direct quotes from your manuscript), or elaborate on this item by providing additional information not in the ms, or briefly explain why the item is not applicable/relevant for your study

"This pragmatic study enrolled participants from an opportunity sample (n=100), comprising eligible candidates."

7b) When applicable, explanation of any interim analyses and stopping guidelines

Does your paper address CONSORT subitem 7b? *

Copy and paste relevant sections from the manuscript (include quotes in quotation marks "like this" to indicate direct quotes from your manuscript), or elaborate on this item by providing additional information not in the ms, or briefly explain why the item is not applicable/relevant for your study

Not applicable as this was a feasibility study

8a) Method used to generate the random allocation sequence

NPT: When applicable, how care providers were allocated to each trial group

Does your paper address CONSORT subitem 8a? *

Copy and paste relevant sections from the manuscript (include quotes in quotation marks "like this" to indicate direct quotes from your manuscript), or elaborate on this item by providing additional information not in the ms, or briefly explain why the item is not applicable/relevant for your study

not applicable, all participants were assigned the same intervention as it was a feasibility/ pilot trial

8b) Type of randomisation; details of any restriction (such as blocking and block size)

Does your paper address CONSORT subitem 8b? *

Copy and paste relevant sections from the manuscript (include quotes in quotation marks "like this" to indicate direct quotes from your manuscript), or elaborate on this item by providing additional information not in the ms, or briefly explain why the item is not applicable/relevant for your study

not applicable, all participants were assigned the same intervention as it was a feasibility/ pilot trial

9) Mechanism used to implement the random allocation sequence (such as sequentially numbered containers), describing any steps taken to conceal the sequence until interventions were assigned

Does your paper address CONSORT subitem 9? *

Copy and paste relevant sections from the manuscript (include quotes in quotation marks "like this" to indicate direct quotes from your manuscript), or elaborate on this item by providing additional information not in the ms, or briefly explain why the item is not applicable/relevant for your study

not applicable, all participants were assigned the same intervention as it was a feasibility/ pilot trial

10) Who generated the random allocation sequence, who enrolled participants, and who assigned participants to interventions

Does your paper address CONSORT subitem 10? *

Copy and paste relevant sections from the manuscript (include quotes in quotation marks "like this" to indicate direct quotes from your manuscript), or elaborate on this item by providing additional information not in the ms, or briefly explain why the item is not applicable/relevant for your study

not applicable, all participants were assigned the same intervention as it was a feasibility/ pilot trial

11a) If done, who was blinded after assignment to interventions (for example, participants, care providers, those assessing outcomes) and how

NPT: Whether or not administering co-interventions were blinded to group assignment

11a-i) Specify who was blinded, and who wasn't

Specify who was blinded, and who wasn't. Usually, in web-based trials it is not possible to blind the participants [1, 3] (this should be clearly acknowledged), but it may be possible to blind outcome assessors, those doing data analysis or those administering co-interventions (if any).

	1	2	3	4	5	
subitem not at all important	(•)	○	○	○	○	essential

Clear selection

Does your paper address subitem 11a-i? *

Copy and paste relevant sections from the manuscript (include quotes in quotation marks "like this" to indicate direct quotes from your manuscript), or elaborate on this item by providing additional information not in the ms, or briefly explain why the item is not applicable/relevant for your study

not applicable, all participants were assigned the same intervention as it was a feasibility/ pilot trial

11a-ii) Discuss e.g., whether participants knew which intervention was the "intervention of interest" and which one was the "comparator"

Informed consent procedures (4a-ii) can create biases and certain expectations - discuss e.g., whether participants knew which intervention was the "intervention of interest" and which one was the "comparator".

	1	2	3	4	5	
subitem not at all important	◉	○	○	○	○	essential

Clear selection

Does your paper address subitem 11a-ii?

Copy and paste relevant sections from the manuscript (include quotes in quotation marks "like this" to indicate direct quotes from your manuscript), or elaborate on this item by providing additional information not in the ms, or briefly explain why the item is not applicable/relevant for your study

not applicable, all participants were assigned the same intervention as it was a feasibility/ pilot trial

11b) If relevant, description of the similarity of interventions

(this item is usually not relevant for ehealth trials as it refers to similarity of a placebo or sham intervention to a active medication/intervention)

Does your paper address CONSORT subitem 11b? *

Copy and paste relevant sections from the manuscript (include quotes in quotation marks "like this" to indicate direct quotes from your manuscript), or elaborate on this item by providing additional information not in the ms, or briefly explain why the item is not applicable/relevant for your study

not applicable - only one intervention

12a) Statistical methods used to compare groups for primary and secondary outcomes

NPT: When applicable, details of whether and how the clustering by care providers or centers was addressed

Does your paper address CONSORT subitem 12a? *

Copy and paste relevant sections from the manuscript (include quotes in quotation marks "like this" to indicate direct quotes from your manuscript), or elaborate on this item by providing additional information not in the ms, or briefly explain why the item is not applicable/relevant for your study

The study was not powered to perform inferential statistical analyses, and so to signal efficacy, we report pre- and post program mean differences and confidence intervals for scores on key outcome measures

12a-i) Imputation techniques to deal with attrition / missing values

Imputation techniques to deal with attrition / missing values: Not all participants will use the intervention/comparator as intended and attrition is typically high in ehealth trials. Specify how participants who did not use the application or dropped out from the trial were treated in the statistical analysis (a complete case analysis is strongly discouraged, and simple imputation techniques such as LOCF may also be problematic [4]).

	1	2	3	4	5	
subitem not at all important	○	○	○	○	◉	essential

Clear selection

Does your paper address subitem 12a-i? *

Copy and paste relevant sections from the manuscript (include quotes in quotation marks "like this" to indicate direct quotes from your manuscript), or elaborate on this item by providing additional information not in the ms, or briefly explain why the item is not applicable/relevant for your study

"responses were made mandatory to avoid missing data"

12b) Methods for additional analyses, such as subgroup analyses and adjusted analyses

Does your paper address CONSORT subitem 12b? *

Copy and paste relevant sections from the manuscript (include quotes in quotation marks "like this" to indicate direct quotes from your manuscript), or elaborate on this item by providing additional information not in the ms, or briefly explain why the item is not applicable/relevant for your study

"Assessment of bias: programme completers versus programme non-completers at baseline
Fifteen participants (n=15) were categorised as 'non-completers' of the Hope Programme (i.e., completing <5 of the 8 sessions). Only four of these participants (n=4/15, 27%) returned both follow-up questionnaires, which was insufficient for meaningful analysis. Assessment of bias was therefore only conducted using baseline data. Potential differences at baseline between non-completers (<5 sessions; n=15) and completers (≥5 sessions; n=42) were explored using descriptive statistics. There were no obvious differences between non-completers and completers in Age (median (IQR) 65.50 (8.75) versus 66.00 (6.50) years respectively); or Index of Multiple Deprivation (7.00 (3.00) versus 7.00 (2.25)). There were slight differences between non-completers and completers in gender (50% versus 31% 'Male'); ethnicity (93% versus 100% 'White'); disability (53% versus 62% 'Yes'); Employment (54% versus 20% 'In paid work – full time' plus 'In paid work – part time); Joint replacement (47% versus 67% 'Knee'); and Waiting time (7.00 (12.00) versus 6.00 (11.00) months) On average, non-completers had slightly greater disease severity. For example, non-completers reported more pain (median (IQR) NPRS = 7.00 (2.00) versus 6.00 (2.00) respectively; had a higher total WOMAC score (60.00 (31.00) versus 49.00 (19.50)); and a lower EQ-5D index value (0.448 (0.548) versus 0.636 (0.450))."

X26) REB/IRB Approval and Ethical Considerations [recommended as subheading under "Methods"] (not a CONSORT item)

X26-i) Comment on ethics committee approval

	1	2	3	4	5	
subitem not at all important	○	○	○	○	◉	essential

Clear selection

Does your paper address subitem X26-i?

Copy and paste relevant sections from the manuscript (include quotes in quotation marks "like this" to indicate direct quotes from your manuscript), or elaborate on this item by providing additional information not in the ms, or briefly explain why the item is not applicable/relevant for your study

"The user requirements research undertaken by Coventry University received ethical approval from the Coventry University Research Ethics Committee (P151751). The research and evaluation activity has also received approval from Coventry University (P106036) and as an amendment to a pre-exiting HOPE evaluation from the HRA and HCRW (IRAS project ID. 283172)"

x26-ii) Outline informed consent procedures

Outline informed consent procedures e.g., if consent was obtained offline or online (how? Checkbox, etc.?), and what information was provided (see 4a-ii). See [6] for some items to be included in informed consent documents.

	1	2	3	4	5	
subitem not at all important	○	○	○	○	◉	essential

Clear selection

Does your paper address subitem X26-ii?

Copy and paste relevant sections from the manuscript (include quotes in quotation marks "like this" to indicate direct quotes from your manuscript), or elaborate on this item by providing additional information not in the ms, or briefly explain why the item is not applicable/relevant for your study

1. I confirm that I have read the information sheet for the above study. I have had the opportunity to consider the information, ask questions and have had these answered satisfactorily.
o Yes
o No

2. I confirm that I am aged 18 years or over.
o Yes
o No

3. I understand that all the information I provide will be treated in confidence.
o Yes
o No

4. I understand that you will be collecting the following data from me: basic sociodemographic information e.g. postcode, age, gender, MSK condition,
o Yes
o No

5. I understand that the information collected about me will be used in an anonymised form for scientific publications and presentations and to support other research in the future, and may be shared anonymously with other researchers.
o Yes
o No

6. I agree to the use of my anonymised direct quotes for reports and publications
o Yes
o No

7. I understand that the information held and maintained by Hope For The Community and Coventry University may be used to help contact me
o Yes
o No

8. I am happy for the session to be video recorded
Yes
No

9. I am happy for the session to be audio recorded
Yes
No

10. I am happy for still images to be used for dissemination purposes
Yes
No

11. I agree to take part in the study
o Yes
o No

X26-iii) Safety and security procedures

Safety and security procedures, incl. privacy considerations, and any steps taken to reduce the likelihood or detection of harm (e.g., education and training, availability of a hotline)

	1	2	3	4	5	
subitem not at all important	○	○	○	○	◉	essential

Clear selection

Does your paper address subitem X26-iii?

Copy and paste relevant sections from the manuscript (include quotes in quotation marks "like this" to indicate direct quotes from your manuscript), or elaborate on this item by providing additional information not in the ms, or briefly explain why the item is not applicable/relevant for your study

Facilitators were trained to QISMET standards

RESULTS

13a) For each group, the numbers of participants who were randomly assigned, received intended treatment, and were analysed for the primary outcome

NPT: The number of care providers or centers performing the intervention in each group and the number of patients treated by each care provider in each center

Does your paper address CONSORT subitem 13a? *

Copy and paste relevant sections from the manuscript (include quotes in quotation marks "like this" to indicate direct quotes from your manuscript), or elaborate on this item by providing additional information not in the ms, or briefly explain why the item is not applicable/relevant for your study

"One hundred participants enrolled on the two Hope Programmes (n=59 and n=41). Of these, n=57 (57%) consented to take part in the evaluation and returned the baseline questionnaire (n=39 Hope Programme 1 and n=18 Hope Programme 2). Forty-two participants (n=42/57, 73.7%) accessed ≥5 of the 8 sessions and were considered

13b) For each group, losses and exclusions after randomisation, together with reasons

Does your paper address CONSORT subitem 13b? (NOTE: Preferably, this is shown in a CONSORT flow diagram) *

Copy and paste relevant sections from the manuscript (include quotes in quotation marks "like this" to indicate direct quotes from your manuscript), or elaborate on this item by providing additional information not in the ms, or briefly explain why the item is not applicable/relevant for your study

A CONSORT flow diagram is provided in the manucsript

13b-i) Attrition diagram

Strongly recommended: An attrition diagram (e.g., proportion of participants still logging in or using the intervention/comparator in each group plotted over time, similar to a survival curve) or other figures or tables demonstrating usage/dose/engagement.

	1	2	3	4	5	
subitem not at all important	○	○	○	◉	○	essential

Clear selection

Does your paper address subitem 13b-i?

Copy and paste relevant sections from the manuscript or cite the figure number if applicable (include quotes in quotation marks "like this" to indicate direct quotes from your manuscript), or elaborate on this item by providing additional information not in the ms, or briefly explain why the item is not applicable/relevant for your study

"Forty-two participants (n=42/57, 73.7%) accessed ≥5 of the 8 sessions and were considered ‘programme completers. "

14a) Dates defining the periods of recruitment and follow-up

Does your paper address CONSORT subitem 14a? *

Copy and paste relevant sections from the manuscript (include quotes in quotation marks "like this" to indicate direct quotes from your manuscript), or elaborate on this item by providing additional information not in the ms, or briefly explain why the item is not applicable/relevant for your study

"Following completion of the pre Hope Programme survey, participants were given access to the Hope Programme (start date 13th or 27th July 2023) "

14a-i) Indicate if critical “secular events” fell into the study period

Indicate if critical “secular events” fell into the study period, e.g., significant changes in Internet resources available or “changes in computer hardware or Internet delivery resources”

	1	2	3	4	5	
subitem not at all important	○	●	○	○	○	essential

Clear selection

Does your paper address subitem 14a-i?

Copy and paste relevant sections from the manuscript (include quotes in quotation marks "like this" to indicate direct quotes from your manuscript), or elaborate on this item by providing additional information not in the ms, or briefly explain why the item is not applicable/relevant for your study

There were no secular events to report.

14b) Why the trial ended or was stopped (early)

Does your paper address CONSORT subitem 14b? *

Copy and paste relevant sections from the manuscript (include quotes in quotation marks "like this" to indicate direct quotes from your manuscript), or elaborate on this item by providing additional information not in the ms, or briefly explain why the item is not applicable/relevant for your study

The trial continued to the end, as planned.

15) A table showing baseline demographic and clinical characteristics for each group

NPT: When applicable, a description of care providers (case volume, qualification, expertise, etc.) and centers (volume) in each group

Does your paper address CONSORT subitem 15? *

Copy and paste relevant sections from the manuscript (include quotes in quotation marks "like this" to indicate direct quotes from your manuscript), or elaborate on this item by providing additional information not in the ms, or briefly explain why the item is not applicable/relevant for your study

"Table 1. Participant characteristics (n=39)"

15-i) Report demographics associated with digital divide issues

In ehealth trials it is particularly important to report demographics associated with digital divide issues, such as age, education, gender, social-economic status, computer/ Internet/ehealth literacy of the participants, if known.

	1	2	3	4	5	
subitem not at all important	○	○	○	●	○	essential

Clear selection

Does your paper address subitem 15-i? *

Copy and paste relevant sections from the manuscript (include quotes in quotation marks "like this" to indicate direct quotes from your manuscript), or elaborate on this item by providing additional information not in the ms, or briefly explain why the item is not applicable/relevant for your study

Please see Table 1.
"All participants identified as 'White - English/Welsh/Scottish/Northern Irish/British' ethnicity and described English as their first language (both n=39/39, 100%). One-third of participants were male (n=13/39; 33%) and two-thirds were female (n=26/39; 67%). Age was only reported by twenty-one participants (n=21/39; 54%), with a median (IQR) age of 66.0 (6.5) years, and the majority of participants were retired (n=23/39; 59%). A third of all participants (n=13/39; 33%) were listed for hip replacement surgery and two-thirds (n=26/39; 67%) for knee surgery. The median Index of Multiple Deprivation was 7.00 (2.25) and the median time on the waiting list for surgery was 6.00 (11.00) months.

Twenty-four participants (n=24/39; 62%) considered themselves to have a disability, with nine participants (n=9/39; 23%) declaring that day to day activities were 'limited a little', and fifteen (n=15/39; 39%) reporting that day to day activities were 'limited a lot'. Seven participants (n=7/39; 18%) reported more than one specific type of disability (see Table 1)."

16) For each group, number of participants (denominator) included in each analysis and whether the analysis was by original assigned groups

16-i) Report multiple “denominators” and provide definitions

Report multiple “denominators” and provide definitions: Report N’s (and effect sizes) “across a range of study participation [and use] thresholds” [1], e.g., N exposed, N consented, N used more than x times, N used more than y weeks, N participants “used” the intervention/comparator at specific pre-defined time points of interest (in absolute and relative numbers per group). Always clearly define “use” of the intervention.

	1	2	3	4	5	
subitem not at all important	○	○	○	◉	○	essential

Clear selection

Does your paper address subitem 16-i? *

Copy and paste relevant sections from the manuscript (include quotes in quotation marks "like this" to indicate direct quotes from your manuscript), or elaborate on this item by providing additional information not in the ms, or briefly explain why the item is not applicable/relevant for your study

"Almost three-quarters of the participants completed all 8 sessions (29/39; 74%), with five participants completing 7 sessions (n=5/39; 13%), one completing 6 sessions (n=1/39; 3%) and a further four participants completing 5 sessions (n=4/39; 10%). All 39 (100%) participants used the personalised exercise programme."

16-ii) Primary analysis should be intent-to-treat

Primary analysis should be intent-to-treat, secondary analyses could include comparing only “users”, with the appropriate caveats that this is no longer a randomized sample (see 18-i).

	1	2	3	4	5	
subitem not at all important	◉	○	○	○	○	essential

Clear selection

Does your paper address subitem 16-ii?

Copy and paste relevant sections from the manuscript (include quotes in quotation marks "like this" to indicate direct quotes from your manuscript), or elaborate on this item by providing additional information not in the ms, or briefly explain why the item is not applicable/relevant for your study

Not applicable with current trial
"Future efficacy trials will employ intention-to-treat (ITT) analysis. "

17a) For each primary and secondary outcome, results for each group, and the estimated effect size and its precision (such as 95% confidence interval)

Does your paper address CONSORT subitem 17a? *

Copy and paste relevant sections from the manuscript (include quotes in quotation marks "like this" to indicate direct quotes from your manuscript), or elaborate on this item by providing additional information not in the ms, or briefly explain why the item is not applicable/relevant for your study

Effect sizes were not reported for this feasibility study. Outcomes are reported as n and percentages, mean scores with standard deviations, mean differences with 95% CIs. Please

17a-i) Presentation of process outcomes such as metrics of use and intensity of use

In addition to primary/secondary (clinical) outcomes, the presentation of process outcomes such as metrics of use and intensity of use (dose, exposure) and their operational definitions is critical. This does not only refer to metrics of attrition (13-b) (often a binary variable), but also to more continuous exposure metrics such as "average session length". These must be accompanied by a technical description how a metric like a "session" is defined (e.g., timeout after idle time) [1] (report under item 6a).

	1	2	3	4	5	
subitem not at all important	○	○	○	○	●	essential

Clear selection

Does your paper address subitem 17a-i?

Copy and paste relevant sections from the manuscript (include quotes in quotation marks "like this" to indicate direct quotes from your manuscript), or elaborate on this item by providing additional information not in the ms, or briefly explain why the item is not applicable/relevant for your study

"The top 5 bookmarked content/activities was 1. exercise programme; 2 relaxed breathing; 3 mindfulness meditation; 4 compassionate approach to pain; 5 Cognitive diffusion

17b) For binary outcomes, presentation of both absolute and relative effect sizes is recommended

Does your paper address CONSORT subitem 17b? *

Copy and paste relevant sections from the manuscript (include quotes in quotation marks "like this" to indicate direct quotes from your manuscript), or elaborate on this item by providing additional information not in the ms, or briefly explain why the item is not applicable/relevant for your study

Not applicable for this study.

18) Results of any other analyses performed, including subgroup analyses and adjusted analyses, distinguishing pre-specified from exploratory

Does your paper address CONSORT subitem 18? *

Copy and paste relevant sections from the manuscript (include quotes in quotation marks "like this" to indicate direct quotes from your manuscript), or elaborate on this item by providing additional information not in the ms, or briefly explain why the item is not applicable/relevant for your study

"Whilst the study was not powered to detect statistically significant changes in outcomes between timepoints, non-parametric Friedman and post-hoc Wilcoxon Signed Rank inferential tests were used to explore changes over time between baseline, post Hope Programme (8 weeks) and 6 months follow-up. All analyses were performed using IBM SPSS v28. The level of statistical significance was set at P<.05. Textual responses to the question about surgery preparedness at the 6 months follow up survey are summarised to illustrate the quantitative findings."

18-i) Subgroup analysis of comparing only users

A subgroup analysis of comparing only users is not uncommon in ehealth trials, but if done, it must be stressed that this is a self-selected sample and no longer an unbiased sample from a randomized trial (see 16-iii).

	1	2	3	4	5	
subitem not at all important	○	○	○	◉	○	essential

Clear selection

Does your paper address subitem 18-i?

Copy and paste relevant sections from the manuscript (include quotes in quotation marks "like this" to indicate direct quotes from your manuscript), or elaborate on this item by providing additional information not in the ms, or briefly explain why the item is not applicable/relevant for your study

"Ancillary analyses:
Assessment of bias: programme completers versus programme non-completers at baseline
Fifteen participants (n=15) were categorised as 'non-completers' of the Hope Programme (i.e., completing <5 of the 8 sessions). Only four of these participants (n=4/15, 27%) returned both follow-up questionnaires, which was insufficient for meaningful analysis. Assessment of bias was therefore only conducted using baseline data. Potential differences at baseline between non-completers (<5 sessions; n=15) and completers (≥5 sessions; n=42) were explored using descriptive statistics. There were no obvious differences between non-completers and completers in Age (median (IQR) 65.50 (8.75) versus 66.00 (6.50) years respectively); or Index of Multiple Deprivation (7.00 (3.00) versus 7.00 (2.25)). There were slight differences between non-completers and completers in gender (50% versus 31% 'Male'); ethnicity (93% versus 100% 'White'); disability (53% versus 62% 'Yes'); Employment (54% versus 20% 'In paid work – full time' plus 'In paid work – part time); Joint replacement (47% versus 67% 'Knee'); and Waiting time (7.00 (12.00) versus 6.00 (11.00) months) On average, non-completers had slightly greater disease severity. For example, non-completers reported more pain (median (IQR) NPRS = 7.00 (2.00) versus 6.00 (2.00) respectively; had a higher total WOMAC score (60.00 (31.00) versus 49.00 (19.50)); and a lower EQ-5D index value (0.448 (0.548) versus 0.636 (0.450))."

19) All important harms or unintended effects in each group
(for specific guidance see CONSORT for harms)

Does your paper address CONSORT subitem 19? *

Copy and paste relevant sections from the manuscript (include quotes in quotation marks "like this" to indicate direct quotes from your manuscript), or elaborate on this item by providing additional information not in the ms, or briefly explain why the item is not applicable/relevant for your study

"Harms
No harms or unintended consequences were reported during the study."

19-i) Include privacy breaches, technical problems

Include privacy breaches, technical problems. This does not only include physical “harm” to participants, but also incidents such as perceived or real privacy breaches [1], technical problems, and other unexpected/unintended incidents. “Unintended effects” also includes unintended positive effects [2].

	1	2	3	4	5	
subitem not at all important	○	○	○	◉	○	essential

Clear selection

Does your paper address subitem 19-i?

Copy and paste relevant sections from the manuscript (include quotes in quotation marks "like this" to indicate direct quotes from your manuscript), or elaborate on this item by providing additional information not in the ms, or briefly explain why the item is not applicable/relevant for your study

No privacy breaches or technical problems to report.

19-ii) Include qualitative feedback from participants or observations from staff/ researchers

Include qualitative feedback from participants or observations from staff/researchers, if available, on strengths and shortcomings of the application, especially if they point to unintended/unexpected effects or uses. This includes (if available) reasons for why people did or did not use the application as intended by the developers.

	1	2	3	4	5	
subitem not at all important	○	○	○	○	◉	essential

Clear selection

Does your paper address subitem 19-ii?

Copy and paste relevant sections from the manuscript (include quotes in quotation marks "like this" to indicate direct quotes from your manuscript), or elaborate on this item by providing additional information not in the ms, or briefly explain why the item is not applicable/relevant for your study

"Satisfaction feedback
All 39 participants provided reasons why they thought they agreed or disagreed why they thought the Hope Programme helped them prepare for surgery. The textual feedback was generally positive. The findings are presented under four headings. personalised exercise; physical and mental preparation; peer support; nothing new. Participant id numbers between 1-14 are those that were still waiting for surgery at 6 months follow up and id numbers between 15-39 are participants who had had surgery.
Personalised exercise
The programme offered exercises that helped patients improve their physical condition and overall preparedness for surgery.
"I have coping strategies and light exercises" (id3)
"Targeted exercises" (id22)
"Better exercised and with better muscle definition" (id19)
"The exercise was good" (id31)
"It gave me some exercises to prepare for surgery." (id29)
"It encouraged me to do the preparation exercises and helped lift my mood when needed." (id20)
Physical and mental preparation
Patients found the programme beneficial for preparing both physically and mentally for surgery. It provided information and insight about what to expect before and after surgery, helping to manage pain, reduce anxiety, increase hope and plan for the future.

"Doing something is better than doing nothing. Trying to maintain what I have has had some benefit, maintaining what I had. And knowledge about the operation, that helped." (id25)
"The programme gave me an insight into what to expect during and after the procedure." (id18)
"I found the information useful and the relaxation techniques particularly helpful." (id38)
"The information given was clear about the future after the operation" (id7)
"Not knowing when surgery was likely to happen The Hope Programme gave me something to think about." (id26)
"I feel I manage pain better even if it becomes more painful." (id8)
I knew so much about what to expect, and I learned techniques to calm any anxiety." (id33)
"I had done exercises, had useful info and mentally prepared for what was going to happen." (id41)
"Both Hope and the hospital teams helped in reducing my anxieties. It also helped it was my 2nd knee surgery." (id21)
"Felt more relaxed" (id24)
"Put me at rest" (id34)
"I was able to look forward to the future and plan my days in a hopeful and useful way." (id36)

Peer support
Connecting with others who have arthritis and are waiting for surgery made patients feel less isolated. The programme offered a platform for discussing shared challenges, such as surgery delays and recovery expectations, fostering a sense of community among participants. Participants valued the emotional support they received through the programme. Sharing experiences with others who were undergoing similar surgeries provided comfort, while insights into the surgical process helped ease fears.
"I have felt supported & informed more about the operation" (id2)
"Hearing what other arthritis sufferers are going through made you feel that you are not

"Hearing what other arthritis sufferers are going through made you feel that you are not alone in dealing with the pain" (id1)
"The Hope Programme gave me the opportunity to share my thoughts/fears with others who had either had their joint surgery or were waiting to undergo it." (id17)
"Useful advice and support." (id4)

Nothing new
A few participants found that the programme covered what they already knew or already had a positive mind set

"I haven't found out anything new" (id9)
"Apart from the exercises, it didn't do anything" (id32)
"I already had a very positive view of how to deal with the issues arising from my arthritis (id10)"
"Some sections helped although some of it nothing new. Maybe a bit on recovery rate, what you can do and when, how to move about for normal daily routine. (id12)"

DISCUSSION

22) Interpretation consistent with results, balancing benefits and harms, and considering other relevant evidence

NPT: In addition, take into account the choice of the comparator, lack of or partial blinding, and unequal expertise of care providers or centers in each group

22-i) Restate study questions and summarize the answers suggested by the data, starting with primary outcomes and process outcomes (use)

Restate study questions and summarize the answers suggested by the data, starting with primary outcomes and process outcomes (use).

	1	2	3	4	5	
subitem not at all important	○	○	○	○	◉	essential

Clear selection

Does your paper address subitem 22-i? *

Copy and paste relevant sections from the manuscript (include quotes in quotation marks "like this" to indicate direct quotes from your manuscript), or elaborate on this item by providing additional information not in the ms, or briefly explain why the item is not applicable/relevant for your study

"This study evaluated the Hope Programme, a digital self-management intervention designed to support patients awaiting hip and knee replacement surgery. Results from 39 participants suggested potential improvements in self-efficacy, pain, health status, and mental wellbeing over six months. Most participants felt better prepared for surgery, and the programme was generally rated positively for usability and satisfaction. "

22-ii) Highlight unanswered new questions, suggest future research

Highlight unanswered new questions, suggest future research.

	1	2	3	4	5	
subitem not at all important	○	○	○	○	◉	essential

Clear selection

Does your paper address subitem 22-ii?

Copy and paste relevant sections from the manuscript (include quotes in quotation marks "like this" to indicate direct quotes from your manuscript), or elaborate on this item by providing additional information not in the ms, or briefly explain why the item is not applicable/relevant for your study

"A future definitive RCT should be appropriately powered to directly compare an intervention group (i.e. the Hope Programme) against an appropriate control group (i.e. treatment as usual). Sub-group analysis should compare PROMs in those who have had, or are still awaiting, surgery at 6-month follow-up. Such a design would help to distinguish the effects of the intervention and the effects of surgery."

20) Trial limitations, addressing sources of potential bias, imprecision, and, if relevant, multiplicity of analyses

20-i) Typical limitations in ehealth trials

Typical limitations in ehealth trials: Participants in ehealth trials are rarely blinded. Ehealth trials often look at a multiplicity of outcomes, increasing risk for a Type I error. Discuss biases due to non-use of the intervention/usability issues, biases through informed consent procedures, unexpected events.

	1	2	3	4	5	
subitem not at all important	○	◉	○	○	○	essential

Clear selection

Does your paper address subitem 20-i? *

Copy and paste relevant sections from the manuscript (include quotes in quotation marks "like this" to indicate direct quotes from your manuscript), or elaborate on this item by providing additional information not in the ms, or briefly explain why the item is not applicable/relevant for your study

"The limitations of the study include the small number (16/39) of participants who completed the SUS Scale. It is possible that these participants had a more positive user experience compared to those participants who did not complete the scale. The main stduy limitation is the lack of a control group. Without a control group comparator, it is not possible to directly attribute any change in the PROMs to the Hope Programme. It is important to note that many improvements were not statistically significant, and that the statistical analyses performed were very likely to be underpowered. Further, a recent systematic review of hip arthroplasty prehabilitation interventions suggested that measures such as the WOMAC may not be the most appropriate measure to detect differences and suggest alternative objective measures such as the chair rise test, gait speed, or stair climbing [53]. That review also found that more than eight weeks of prehabilitation was associated with improved outcomes, suggesting that future trials of the Hope Programme should consider extending the length of the intervention. While our completer analysis provides valuable proof-of-concept data, it limits generalizability to real-world implementation, where attrition is typically higher. The baseline differences between completers and non-completers suggest our effect estimates may be optimistic. However, the high follow-up rates (93-98%) provide confidence that attrition bias is less severe than in typical digital health studies. Future randomized controlled trials (RCTs) should combine ITT and per-protocol analyses to distinguish efficacy from effectiveness."

21) Generalisability (external validity, applicability) of the trial findings

NPT: External validity of the trial findings according to the intervention, comparators, patients, and care providers or centers involved in the trial

21-i) Generalizability to other populations

Generalizability to other populations: In particular, discuss generalizability to a general Internet population, outside of a RCT setting, and general patient population, including applicability of the study results for other organizations

	1	2	3	4	5	
subitem not at all important	○	○	○	○	◉	essential

Clear selection

Does your paper address subitem 21-i?

Copy and paste relevant sections from the manuscript (include quotes in quotation marks "like this" to indicate direct quotes from your manuscript), or elaborate on this item by providing additional information not in the ms, or briefly explain why the item is not applicable/relevant for your study

"The limitations of the study include the small number (16/39) of participants who completed the SUS Scale. It is possible that these participants had a more positive user experience compared to those participants who did not complete the scale. The self-selecting nature of recruitment may result in participants who are inherently more inclined to seek assistance or engage in self-help efforts"

21-ii) Discuss if there were elements in the RCT that would be different in a routine application setting

Discuss if there were elements in the RCT that would be different in a routine application setting (e.g., prompts/reminders, more human involvement, training sessions or other co-interventions) and what impact the omission of these elements could have on use, adoption, or outcomes if the intervention is applied outside of a RCT setting.

	1	2	3	4	5	
subitem not at all important	○	○	○	◉	○	essential

Clear selection

Does your paper address subitem 21-ii?

Copy and paste relevant sections from the manuscript (include quotes in quotation marks "like this" to indicate direct quotes from your manuscript), or elaborate on this item by providing additional information not in the ms, or briefly explain why the item is not applicable/relevant for your study

Not applicable to this study

OTHER INFORMATION

23) Registration number and name of trial registry

Does your paper address CONSORT subitem 23? *

Copy and paste relevant sections from the manuscript (include quotes in quotation marks "like this" to indicate direct quotes from your manuscript), or elaborate on this item by providing additional information not in the ms, or briefly explain why the item is not applicable/relevant for your study

This pilot study was not registered with a clinical trials registry.

24) Where the full trial protocol can be accessed, if available

Does your paper address CONSORT subitem 24? *

Cite a Multimedia Appendix, other reference, or copy and paste relevant sections from the manuscript (include quotes in quotation marks "like this" to indicate direct quotes from your manuscript), or elaborate on this item by providing additional information not in the ms, or briefly explain why the item is not applicable/relevant for your study

This pilot study was not registered with a clinical trials registry.

25) Sources of funding and other support (such as supply of drugs), role of funders

Does your paper address CONSORT subitem 25? *

Copy and paste relevant sections from the manuscript (include quotes in quotation marks "like this" to indicate direct quotes from your manuscript), or elaborate on this item by providing additional information not in the ms, or briefly explain why the item is not applicable/relevant for your study

Funding was awarded to Hope 4 The Community (H4C) Community Interest Company from the Small Business Research Initiative Social Ventures competition, run by the UK Research and Innovation as part of the Healthy Ageing Challenge, delivered by Innovate UK.

The user requirements research undertaken by Coventry University received ethical approval from the Coventry University Research Ethics Committee (P151751). The research and evaluation activity has also received approval from Coventry University (P106036) and as an amendment to a pre-exiting HOPE evaluation from the HRA and

X27) Conflicts of Interest (not a CONSORT item)

X27-i) State the relation of the study team towards the system being evaluated

In addition to the usual declaration of interests (financial or otherwise), also state the relation of the study team towards the system being evaluated, i.e., state if the authors/evaluators are distinct from or identical with the developers/sponsors of the intervention.

	1	2	3	4	5	
subitem not at all important	○	○	○	●	○	essential

Clear selection

Does your paper address subitem X27-i?

Copy and paste relevant sections from the manuscript (include quotes in quotation marks "like this" to indicate direct quotes from your manuscript), or elaborate on this item by providing additional information not in the ms, or briefly explain why the item is not applicable/relevant for your study

Andy Turner is co-founder of the HOPE Program and non-executive director of H4C.

About the CONSORT EHEALTH checklist

As a result of using this checklist, did you make changes in your manuscript? *

- ○ yes, major changes
- ○ yes, minor changes
- ◉ no

What were the most important changes you made as a result of using this checklist?

Your answer

How much time did you spend on going through the checklist INCLUDING making changes in your manuscript *

Four hours in total

As a result of using this checklist, do you think your manuscript has improved? *

- ○ yes
- ◉ no
- ○ Other:

Would you like to become involved in the CONSORT EHEALTH group?

This would involve for example becoming involved in participating in a workshop and writing an "Explanation and Elaboration" document

- ○ yes
- ◉ no
- ○ Other:

Clear selection

Any other comments or questions on CONSORT EHEALTH

It takes far too long to complete the checklist . A lot of the information provided on the checklist was duplication of what is presented in the paper - which was prepared in line

STOP - Save this form as PDF before you click submit

To generate a record that you filled in this form, we recommend to generate a PDF of this page (on a Mac, simply select "print" and then select "print as PDF") before you submit it.

When you submit your (revised) paper to JMIR, please upload the PDF as supplementary file.

Don't worry if some text in the textboxes is cut off, as we still have the complete information in our database. Thank you!

Final step: Click submit !

Click submit so we have your answers in our database!

Submit

Clear form

Never submit passwords through Google Forms.

This content is neither created nor endorsed by Google. - Contact form owner - Terms of Service - Privacy Policy

Does this form look suspicious? Report

Google Forms
